# Deep learning-based PET image denoising and reconstruction: a review

**DOI:** 10.1007/s12194-024-00780-3

**Published:** 2024-02-06

**Authors:** Fumio Hashimoto, Yuya Onishi, Kibo Ote, Hideaki Tashima, Andrew J. Reader, Taiga Yamaya

**Affiliations:** 1 Central Research Laboratory, Hamamatsu Photonics K. K, 5000 Hirakuchi, Hamana-Ku, Hamamatsu, 434-8601 Japan; 2 Graduate School of Science and Engineering, Chiba University, 1-33, Yayoicho, Inage-Ku, Chiba, 263-8522 Japan; 3 National Institutes for Quantum Science and Technology, 4-9-1, Anagawa, Inage-Ku, Chiba, 263-8555 Japan; 4 School of Biomedical Engineering and Imaging Sciences, King’s College London, London, SE1 7EH UK

**Keywords:** Positron emission tomography, Deep learning, Image reconstruction, Convolutional neural networks

## Abstract

This review focuses on positron emission tomography (PET) imaging algorithms and traces the evolution of PET image reconstruction methods. First, we provide an overview of conventional PET image reconstruction methods from filtered backprojection through to recent iterative PET image reconstruction algorithms, and then review deep learning methods for PET data up to the latest innovations within three main categories. The first category involves post-processing methods for PET image denoising. The second category comprises direct image reconstruction methods that learn mappings from sinograms to the reconstructed images in an end-to-end manner. The third category comprises iterative reconstruction methods that combine conventional iterative image reconstruction with neural-network enhancement. We discuss future perspectives on PET imaging and deep learning technology.

## Introduction

Deep learning has been a popular topic in the scientific communities for a long time due to its significant impact in many fields [1–3]. A new wave of deep learning has been sweeping the medical imaging field [4–10], and some deep learning techniques, such as image reconstruction and computer-aided diagnosis, have already been implemented in commercial systems [11–14]. In this review, we focus on positron emission tomography (PET) imaging using deep learning in the field of medical imaging [15–24].

PET is a molecular imaging method for visualizing and quantifying the distribution of radioactive tracers labeled with positron-emitting radioisotopes, such as fluorine-18 (^18^F), oxygen-15 (^15^O), nitrogen-13 (^13^N), and carbon-11 (^11^C), administered to living human participants [25]. Thus, PET can observe tracer kinetics; therefore, it is used not only for cancer diagnosis [26, 27] and diagnosis of neurodegenerative diseases, such as Alzheimer's disease [28, 29], but for fundamental research, such as brain function [30, 31]. PET is a unique imaging modality capable of tracking picomole-order molecules; however, image noise is severe compared to other tomographic scanners, such as X-ray computed tomography (CT) because there are fewer counts in the measured data. Image noise degrades quantitative accuracy and lesion detectability, leading to the potential scenario of missed lesions. One straightforward strategy for improving PET image quality (or suppressing PET image noise) is to increase the amount of PET tracer administered to the individual. This is sometimes difficult to actively adopt because of the problem of increased radiation exposure [32] and the limitations in count-rate capabilities of PET scanners. Therefore, there is a demand for noise reduction techniques that do not increase injected dose. It is no exaggeration to state that the development history of PET imaging has been a battle against noise.

In this review, we highlight the algorithms used for PET imaging and systematically describe the history of PET image reconstruction and post-processing denoising algorithms from early analytical methods to the latest advances in deep learning technology. Section 2 describes the basic principles of PET imaging, including PET imaging models, conventional analytical and statistical PET image reconstruction algorithms, and an overview of deep learning-based PET imaging algorithms. Section 3 reviews deep learning for PET image denoising algorithms, and Sects. 4, 5, and 6 review deep learning for direct, iterative, and dynamic PET image reconstruction algorithms, respectively. Finally, we conclude this review by providing future perspectives on PET imaging and deep learning technology.

## Basic principles of PET imaging

**Fig. 1:**
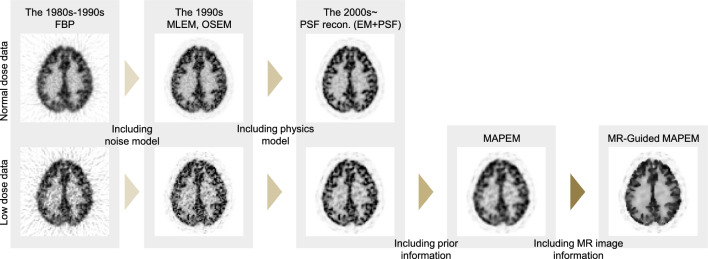
Demonstration of various PET image reconstruction algorithms from the FBP to recent iterative PET image reconstruction algorithms, which were applied to the same simulation data generated from the BrainWeb (https://brainweb.bic.mni.mcgill.ca/brainweb/)

This section briefly reviews the history of PET image reconstruction prior to the advent of deep learning techniques. Figure 1 summarizes the evolution of PET image reconstruction between the 1980s and 2000s.

Between the 1970s and 1980s, researchers developed analytical reconstruction methods, such as the filtered backprojection (FBP) algorithm for tomographic imaging systems, such as X-ray CT and PET [33–35]. FBP is an analytical method that models the relationship between the image and the tomographic measurement data through an integral equation [36] as follows:

1 $$\begin{array}{c}Y\left(r,\phi \right)={\int }_{-\infty }^{\infty }X\left(r{\text{cos}}\phi -s{\text{sin}}\phi ,r{\text{sin}}\phi +s{\text{cos}}\phi \right)ds,\end{array}$$

where $X\left(u,v\right)$ is a two-dimensional (2D) image and $Y\left(r,\phi \right)$ holds 1D projections for each view angles, known as a sinogram. The sinogram is an integral along the $s$-axis of a rotated image by an angle $\phi$. This integral transformation is known as the Radon transform [37] or X-ray transform. The principle behind FBP is the projection slice theorem that shows the relationship between the 2D Fourier transform of $X\left(u,v\right)$ and the 1D Fourier transform of $Y\left(r,\phi \right)$, with respect to *r* as a one-to-one mapping. The most commonly used analytical method is FBP, which is calculated as follows:

2 $$\begin{array}{c}\begin{array}{c}X\left(u,v\right)={\int }_{0}^{\pi }{\left.{Y}_{filtered}\left(r,\phi \right)\right|}_{r=ucos\phi +vsin\phi }d\phi ,\\ {Y}_{filtered}\left(r,\phi \right)={\int }_{-\infty }^{+\infty }G\left(\xi ,\phi \right)\left|\xi \right|{\text{exp}}\left(2\pi i\xi r\right)d\xi \\ G\left(\xi ,\phi \right)={\int }_{-\infty }^{+\infty }Y\left(r,\phi \right){\text{exp}}\left(-2\pi i\xi r\right)dr,\end{array},\end{array}$$

where $i$ is the imaginary unit, $\xi$ is the frequency domain variable, and the high pass filter $\left|\xi \right|$ is called a ramp filter. Although the ramp filter is an analytically derived necessity, enhancing the high-frequency components tends to produce severe noise. Therefore, frequency cutoff techniques and various filters have been developed to reduce high-frequency noise, although they sacrifice spatial resolution. In principle, the analytical method is known for its high speed, linearity, and quantitative accuracy; however, it is susceptible to noise and leads to streak artifacts in low-count situations, as shown in Fig. 1.

Early PET systems had septa inserted between detector rings to shield gamma rays from oblique directions, and measured data to be processed were limited to a 2D plane. In the 1980s, researchers developed 3D PET image reconstruction methods [38–40], and since the 1990s, 3D acquisition has been performed by removing the septa or making them retractable and switchable for 2D and 3D modes [41, 42]. While projections in a range of 0 to 180° for the directions perpendicular to the axial direction form a complete set for reconstruction of a 3D image, the 3D acquisition significantly improved the sensitivity by measuring oblique projections. To fully utilize 3D projection data, image reconstruction methods that consider data redundancy are required. We should note that, in addition, scatter correction is essential for quantification due to the increased scatter components in 3D projection data, contrary to the 2D acquisition where the septa shield most components scattered inside a patient's body. Scatter components are estimated and subtracted from projection data before analytical image reconstruction. There are many studies regarding the estimation and the impact of the scatter [43–49], but they are out of the scope of this article. One of the most used analytical image reconstruction methods for the 3D PET is the 3D reprojection (3DRP) algorithm [39], which includes estimating the missing truncated data in 2D projections in order to apply 3D FBP. The 3D FBP is an extension of 2D FBP to three dimensions, where the Colsher filter [38] is applied to 2D parallel projections for each projection direction parameterized by azimuthal (phi) and co-polar (theta) angles. Note that the set of 2D parallel projections for the co-polar (oblique) angle of 0 is equivalent to the stack of sinograms for 2D FBP (direct sinograms). Then, the filtered 2D parallel projections are back-projected to the image domain. Although the 3D FBP requires projection data without truncation, projections in oblique angles have unmeasured regions against objects inside cylindrical PET scanners. The truncated data are estimated from a 3D image reconstructed as a stack of 2D images by 2D FBP for direct sinograms. The 3DRP method of directly treating 3D projection data was a computational burden for computers in the early 1990s. Therefore, the Fourier rebinning (FORE) method [50, 51] was developed for rebinning 3D projection data to a stack of 2D sinograms, allowing decomposition of the 3D image reconstruction problem into a set of 2D image reconstructions. Not only 2D FBP but iterative 2D image reconstruction methods can be applied following the FORE [52–54]. As a result of the maturation of 3D image reconstruction, modern PET scanners no longer use septa. In recent computers, 3D reconstruction methods have become tractable, and even iterative methods are used in practice.

Between the 1980s and 1990s, iterative reconstruction methods, such as the maximum likelihood expectation maximization (MLEM) algorithm [55–57], were developed to incorporate statistical and physical models into image reconstruction. In the EM iterative method, the relationship between a tomographic image and measured data is modeled through a system of linear equations and Poisson distribution [58] as follows:

3 $$\begin{array}{*{20}c} {y = Poisson \left( {{\varvec{Ax}} + \overline{\user2{b}}} \right),} \\ \end{array}$$

or

4 $$\begin{array}{*{20}c} {y_{i} = Poisson\left( {\mathop \sum \limits_{j = 1}^{J} a_{ij} x_{j} + \overline{b}_{i} } \right),} \\ \end{array}$$

where ${\varvec{x}}={\left({x}_{1},{x}_{2},\cdots ,{x}_{J}\right)}^{T}$ is a vector of voxel values of the image, ${\varvec{y}}={\left({y}_{1},{y}_{2},\cdots ,{y}_{I}\right)}^{T}$ is a vector of sampled values of the projection data, $\overline{{\varvec{b}} }={\left({\overline{b} }_{1},{b}_{2},\cdots ,{\overline{b} }_{I}\right)}^{T}$ is a vector of expected values of background components, such as scatter and random coincidence events, which can be estimated using scatter and randoms modeling methods, and ${\varvec{A}}\in {\mathbb{R}}^{I\times J}$ is a system matrix where each element, and ${a}_{ij},$ is the probability that two gamma rays emitted from $j$-th voxel are detected by $i$-th line-of-response (LOR). The negative log-likelihood function of data ${\varvec{y}}$ under image ${\varvec{x}}$ is defined as follows:

5 $$\begin{array}{*{20}c} {L\left( {{\varvec{y}}{|}{\varvec{x}}} \right) = - \log P\left( {{\varvec{y}}{|}{\varvec{x}}} \right) = C - \mathop \sum \limits_{i = 1}^{I} \left\{ {y_{i} \log \left( {\mathop \sum \limits_{j = 1}^{J} a_{ij} x_{j} + \overline{b}_{i} } \right) - \left( {\mathop \sum \limits_{j = 1}^{J} a_{ij} x_{j} + \overline{b}_{i} } \right)} \right\},} \\ \end{array}$$

where $P\left({\varvec{y}}|{\varvec{x}}\right)$ is the probability of sampling ${\varvec{y}}$ under ${\varvec{x}}$**,** and $C$ is a constant. The MLEM algorithm estimates an image by minimizing (5) using iterative updates as follows:

6 $$\begin{array}{*{20}c} {x_{j}^{{\left( {k + 1} \right)}} = \frac{{x_{j}^{\left( k \right)} }}{{\mathop \sum \nolimits_{i = 1}^{I} a_{ij} }}\mathop \sum \limits_{i = 1}^{I} \frac{{a_{ij} y_{i} }}{{\mathop \sum \nolimits_{{j^{\prime} = 1}}^{J} a_{{ij^{\prime}}} x_{{j^{\prime}}}^{\left( k \right)} + \overline{b}_{i} }},} \\ \end{array}$$

where $k$ denotes the iteration number. The MLEM algorithm achieves better image quality than the FBP algorithm by leveraging a statistical noise model for PET, as shown in Fig. 1. After introducing the MLEM algorithm, the ordered subset expectation maximization (OSEM) algorithm [59], a block iterative reconstruction method that divides the projection data into subsets and updates the image for each subset, was developed as a speed-up method. Furthermore, Tanaka and Kudo proposed a dynamic row action maximum likelihood (DRAMA) algorithm [60, 61]. The DRAMA algorithm contributes to improved convergence speed in the reconstruction process by controlling an optimal relaxation factor deduced by balancing the noise propagation from each subset to the final reconstructed image [61, 62]. We should note that these algorithms can be also applied for 3D PET data and even time-of-flight (TOF) PET data by properly modeling the system matrix.

Between the 1990s and 2000s, iterative reconstruction methods integrating the point-spread function (PSF) were developed for dedicated PET [63–66], as well as whole-body PET/CT [67]. The PSF can be modeled in either projection and/or image space. An example of incorporating an image-space PSF is as follows [64]:

7 $$\begin{array}{*{20}c} {{\varvec{x}}^{{\left( {k + 1} \right)}} = \frac{{{\varvec{x}}^{\left( k \right)} }}{{{\varvec{H}}^{T} {\varvec{A}}^{T} 1}}{\varvec{H}}^{T} {\varvec{A}}^{T} \frac{{\varvec{y}}}{{{\varvec{AHx}}^{\left( k \right)} + \overline{\user2{b}}}},} \\ \end{array}$$

where ${\varvec{H}} \in {\mathbb{R}}^{J \times J}$ is a matrix comprising the PSF kernel in the image space. In Eq. (7), the division and multiplication between vectors are element-wise. Note that Eq. (7) is equivalent to Eq. (6) when ${\varvec{H}}$ is identity matrix. The PSF image reconstruction primarily reduces image noise and enhances contrast as well as improves spatial resolution, as shown in Fig. 1. The PSF kernel increases the correlation between voxels and reduces their variance. From this point of view, the image-space PSF can be considered a variant of the basis function approach [68, 69].

Parallel to the development of statistical and physical model-based iterative reconstructions, maximum a posteriori (MAP) reconstruction methods that incorporate image priors, such as smoothness to maximum likelihood estimation, have been developed [63, 70–75]. The MLEM algorithm exhibits an unfavorable property whereby noise and edge artifacts tend to increase as the iterations progress [76, 77]. Thus, practical solutions involve early stopping of iterations and/or post-smoothing using a Gaussian filter [78]. The MAP reconstruction presents an alternative solution that often achieves a more favorable balance between noise and contrast than the above-mentioned techniques [79]. The posterior probability of image ${\varvec{x}}$ given data ${\varvec{y}}$ is expressed through Bayes’ theorem as follows:

8 $$\begin{array}{*{20}c} {P\left( {{\varvec{x}}{|}{\varvec{y}}} \right) = \frac{{P\left( {{\varvec{y}}{|}{\varvec{x}}} \right)P\left( {\varvec{x}} \right)}}{{P\left( {\varvec{y}} \right)}},} \\ \end{array}$$

where $P\left( {\varvec{x}} \right)$ is the prior probability of image ${\varvec{x}}$. The prior probability is assumed to be the following exponential function called the Gibbs distribution:

9 $$\begin{array}{*{20}c} {P\left( {\varvec{x}} \right) = \frac{1}{Z}exp\left( { - \beta U\left( {\varvec{x}} \right)} \right),} \\ \end{array}$$

where $Z$ is a partition function that makes the sum of the probabilities 1, and $U\left( {\varvec{x}} \right)$ is an energy function designed to be small when the image is correct. The negative log-posterior likelihood is defined as follows:

10 $$\begin{array}{*{20}c} { - \log P\left( {{\varvec{y}}{|}{\varvec{x}}} \right) - \log P\left( {\varvec{x}} \right) = L\left( {{\varvec{y}}{|}{\varvec{x}}} \right) + \beta U\left( {\varvec{x}} \right),} \\ \end{array}$$

where $\beta$ is a hyperparameter that adjusts the influence of the prior distribution. Various MAP estimations are customized based on the selection of the prior distribution in the form of the Gibbs distribution. A commonly used energy function for the Gibbs distribution is as follows:

11 $$\begin{array}{*{20}c} {U\left( {\varvec{x}} \right) = \mathop \sum \limits_{j} \mathop \sum \limits_{{j^{\prime} \in N_{j} }} \omega_{{jj^{\prime}}} V\left( {x_{j} - x_{{j^{\prime}}} } \right),} \\ \end{array}$$

where $V\left( \cdot \right)$ is a potential function, $N_{j}$ is a set of neighboring voxels for the $j$-th voxel, and ${\omega }_{j{j}{\prime}}$ is a weight between neighboring voxels. The weight is typically defined as the inverse of the distance between neighboring voxels. Examples of potential functions include quadratic and relative difference [74], as follows:

12 $$\begin{array}{*{20}c} {\begin{array}{*{20}c} {{\text{Quadratic}}} & {\left( {x_{j} - x_{{j^{\prime}}} } \right)^{2} } \\ {{\text{Relative}} {\text{difference}}} & {\frac{{\left( {x_{j} - x_{{j^{\prime}}} } \right)^{2} }}{{\left( {x_{j} + x_{{j^{\prime}}} } \right) + \gamma \left| {x_{j} - x_{{j^{\prime}}} } \right|}}} \\ \end{array} ,} \\ \end{array}$$

where $\gamma$ is a hyperparameter controlling the shape of relative difference. To minimize negative log-posterior likelihood function, Green’s one-step-late method [72] is commonly used as follows:

13 $$\begin{array}{*{20}c} {x_{j}^{{\left( {k + 1} \right)}} = \frac{{x_{j}^{\left( k \right)} }}{{\mathop \sum \nolimits_{i = 1}^{I} a_{ij} + \beta \left. {\frac{{\partial U\left( {\varvec{x}} \right)}}{{\partial x_{j} }}} \right|_{{{\varvec{x}} = {\varvec{x}}^{\left( k \right)} }} }}\mathop \sum \limits_{i = 1}^{I} \frac{{a_{ij} y_{i} }}{{\mathop \sum \nolimits_{{j^{\prime} = 1}}^{J} a_{{ij^{\prime}}} x_{{j^{\prime}}}^{\left( k \right)} + \overline{b}_{i} }}.} \\ \end{array}$$

In the PET image reconstruction, the use of MAPEM with a quadratic prior provides a smoother image than the MLEM algorithm in low-count situations, as shown in Fig. 1.

With the emergence of PET/CT and PET/magnetic resonance imaging (MRI) scanners, the MAPEM algorithms that incorporate additional anatomical information from CT and MR images [80–84] were also developed. For example, we can incorporate MRI information into MAPEM by setting the weight ${\omega }_{j{j}{\prime}}$ based on the difference between $j$- and ${j}{\prime}$-th voxel values of MRI (as detailed in Sect. 5). As shown in Fig. 1, MR-guided MAPEM can provide images with enhanced smoothness while preserving the organ boundaries.

**Fig. 2:**
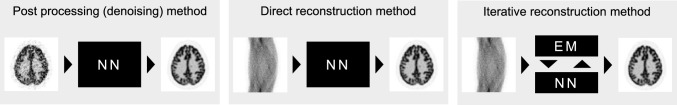
Overview of the deep learning methods for PET data: They are divided into three categories; post-processing (denoising), direct reconstruction, and iterative reconstruction methods using neural networks (NNs)

Currently, the trajectory of PET image reconstruction is undergoing a deeper evolution, propelled by the integration of state-of-the-art deep learning technology in conjunction with computer vision techniques [85–89]. Figure 2 shows a classification of the deep learning methods for PET data in this review, which are strategically categorized into three distinct classes. First, the earliest deep learning methods for PET imaging primarily focused on post-processing for PET image denoising. Notably, these methods do not strictly perform image reconstruction processes. Second, a direct image reconstruction is a data-driven approach to learn a direct mapping from sinogram to PET image using training datasets of sinograms and reconstructed images. Third, an iterative reconstruction is a hybrid approach that utilizes existing image reconstruction combined with neural-network image enhancement. We proceed with more details of deep learning-based PET imaging methods in the following sections.

## Deep learning for PET image denoising

Reconstructed PET images typically exhibit a low signal-to-noise ratio, owing to physical degradation factors and limited statistical counts. Low-dose radiotracers or short-time scans that reduce patient burden accelerate the degradation of PET images, potentially affecting diagnostic accuracy. This remains a major challenge and an effective restoration approach for low-quality PET images is essential. The restoration of PET images is sometimes included as a “reconstruction” process; however, this section focuses on restoration methods by post-processing after reconstruction, distinguishing it from reconstruction that generates images from measurement data.

Noise occurs as the image reconstruction is ill conditioned, such that a small perturbation of the measurement data greatly affects the image with much larger perturbations, as follows:

14 $$\begin{array}{*{20}c} {\hat{\user2{x}} = {\user2{x}} + {\user2{n}}, } \\ \end{array}$$

where $\widehat{{\varvec{x}}}$, ${\varvec{x}}$, and ${\varvec{n}}$ are the degraded PET image, true PET image, and degraded component, respectively. The PET image denoising (or restoration) task is an inverse problem, whereby restoring the original image from a degraded image additively mixed with statistical noise complicated by the image reconstruction process. In recent years, deep learning approaches have been proposed to train the relationship between $\widehat{{\varvec{x}}}$ and ${\varvec{x}}$ using the following minimization problem:

15 $$\begin{array}{*{20}c} {\theta^{*} = \mathop {{\text{argmin}}}\limits_{\theta } E\left( {f\left( {\theta {|}\hat{\user2{x}}} \right);{\varvec{x}}} \right),} \\ \end{array}$$

where $f$ represents a neural-network model with trainable parameters $\theta$, $E$ is a loss function such as a mean-squared error (MSE) or mean absolute error. In general, deep learning-based PET image denoising aims to acquire data-driven nonlinear mapping from low-quality to high-quality PET images. It provides better denoising performance while retaining the spatial resolution and quantitative accuracy compared with classical denoising methods. In this section, we introduce deep learning-based PET image denoising methods based on the power of convolutional neural networks (CNNs) that specialize in image mappings in three ways to be covered below: supervised learning, self-supervised and unsupervised learning, and emerging approaches.

### Supervised learning approach

**Fig. 3:**
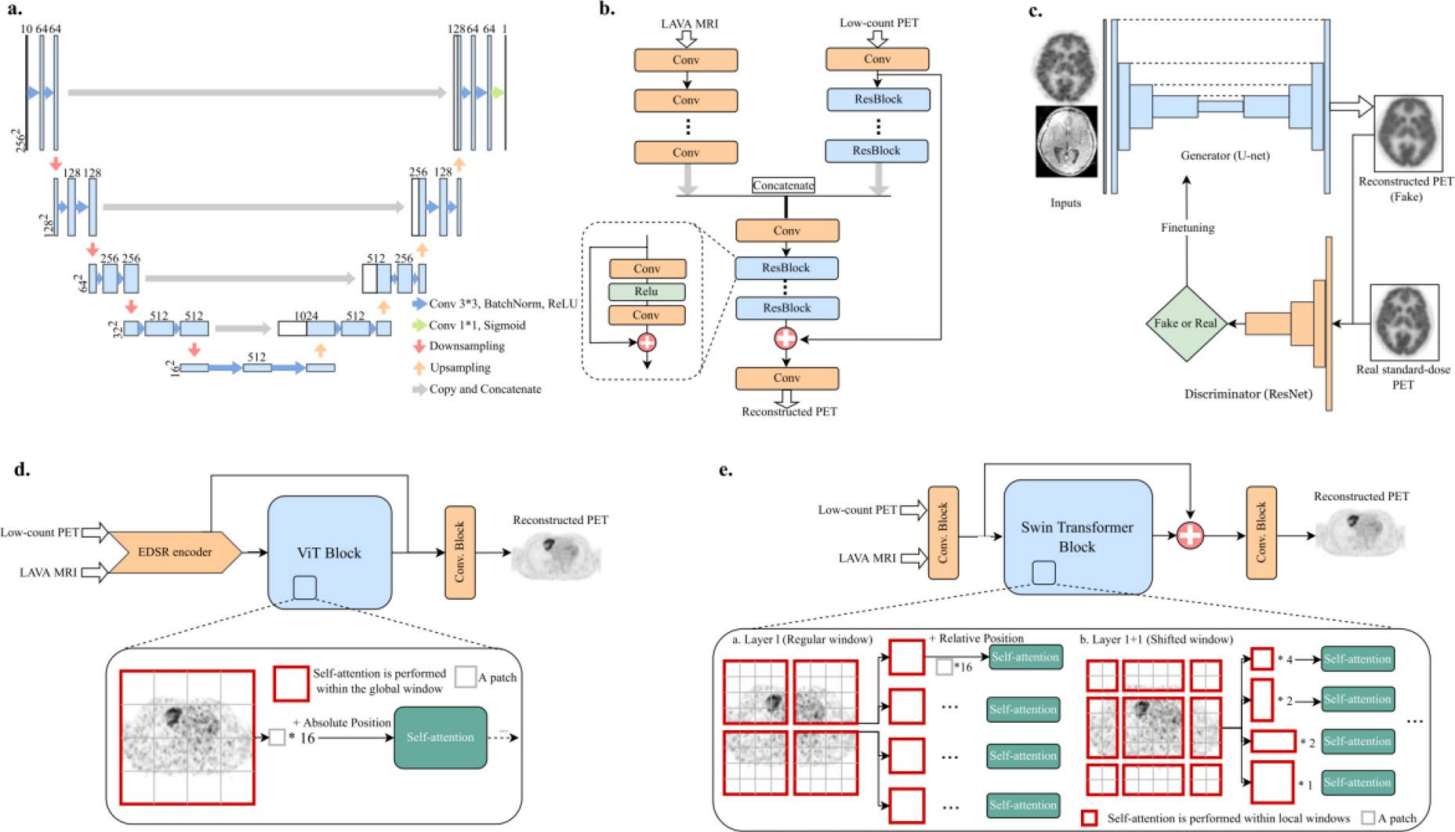
Overview of the various deep learning architectures for PET image denoising. (a) U-Net model. (b) Multi-modal network using anatomical information. (c) GAN model. (d) Vision Transformer (ViT) model. (e) Swin Transformer image restoration network (SwinIR). © 2023 SNCSC. Reprinted with permission from Wang et al. [159]

Supervised learning is an approach used in machine learning to train models based on labeled data. PET image denoising tasks require huge datasets, comprising pairs of high- and low-quality PET images, as shown in Eq. (14). The evolution of deep learning has led to the transformation of shallow CNNs, initially implemented with only a few convolutional layers into architectures with deeper layers. This progress has enabled more potent PET image denoising capabilities, as evidenced by their superior performance [90]. Starting with these successes, CNN architectures have more complex features and have developed into structures specialized for image denoising and medical image processing, as shown in Fig. 3. Among them, the U-Net proposed by Ronneberger et al. [91] and 3D U-Net proposed by Çiçek [92] for semantic segmentation are widely used for PET image denoising [93–95]. A typical U-Net architecture consists of a contracting path to capture the context from the input image and a symmetric expanding path that up-samples the extracted feature map. In addition, the U-Net architecture introduces skip connections that pass the feature maps at each resolution of the contracting path to the expanding path. Residual learning [96] has also been proposed, in which the noise component contained in the image is output based on the idea that it is easier to leave only latent noise rather than retain the complex visual features of the PET image in the hidden layer [97–100]. Perceptual loss, which is based on high-level feature representations extracted from a pre-trained VGG16 on ImageNet, has been shown to improve the visual quality of PET images compared to general loss functions, such as the MSE [101]. Recently, the widespread use of PET/CT or PET/MR scanners has facilitated the simultaneous acquisition of functional and anatomical images. Therefore, PET image denoising is also performed by combining multimodal anatomical information, such as CT [102, 103] or MR images [104–112], thereby achieving superior denoising performance compared with PET alone.

**Fig. 4:**
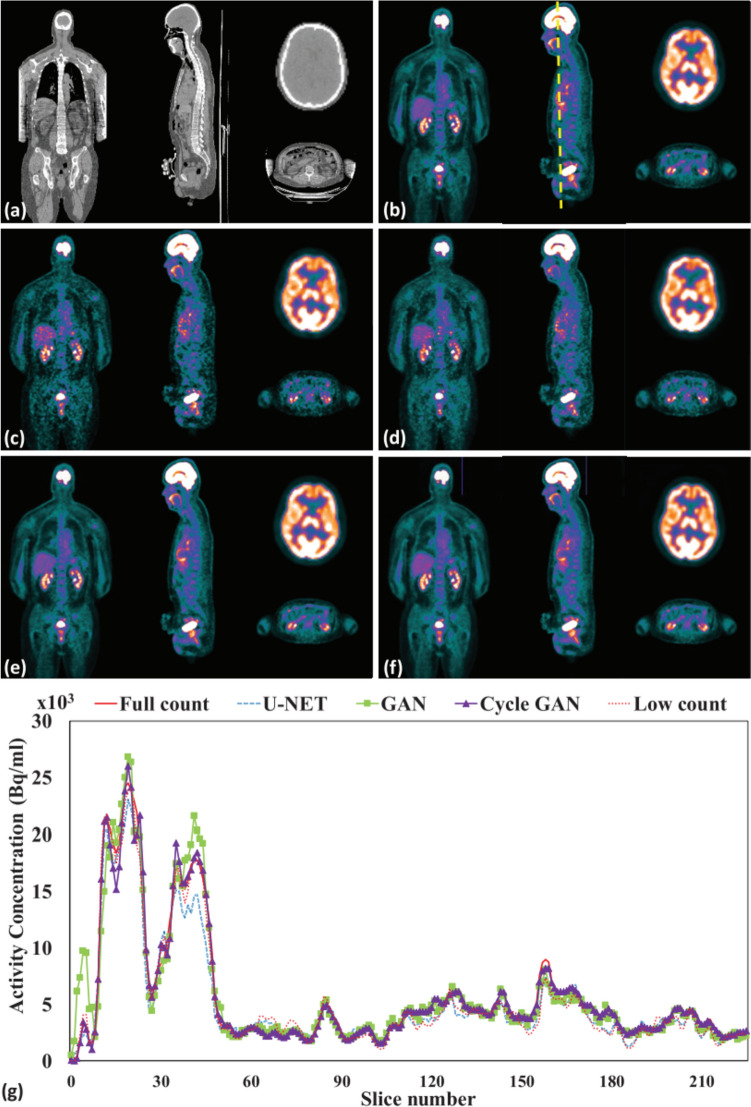
Examples of the denoised whole body.^18^F-FDG PET images by supervised learning approaches. Sample images showing (a) CT, (b) full-count PET, (c) low-count PET, and denoised PET images corresponding to the (d) U-Net, (e) GAN, and (f) CycleGAN. (g) Line profiles in sagittal section. © 2019 IOP Publishing. Reprinted with permission from Lei et al. [127]

The advent of generative adversarial networks (GAN) has led to breakthroughs in the field of image generation [113]. The GAN consists of two competing neural networks: a generator and a discriminator. In addition to adopting a network, such as a U-Net, which is capable of image-to-image translation as a generator, it can be regarded as a training method that considers the adversarial loss based on the output from the discriminator. GAN training proceeds such that the label data are no longer distinguishable from the output images of the CNN, thereby synthesizing denoised PET images with less spatial blur and better visual quality [114–116]. Common models for denoising by GANs include Conditional GAN [117] and Pix2Pix [118], while incorporating various network structures [119, 120] and additional loss functions, such as least squares [121, 122], task-specific perceptual loss [123], pixelwise loss [124], and Wasserstein distance with a gradient penalty [125], have all been reported to improve denoising performance. CycleGAN is a method that consists of two generator and discriminator pairs with cycle consistency loss [126], which can train denoising tasks without a corresponding direct pairing between the degraded and original PET images, which is conventionally essential (Fig. 4) [127–131].

### Self-supervised and unsupervised learning approaches

Collecting a large number of high-quality PET images for supervised learning is particularly difficult in clinical practice. Furthermore, the generalization performance for various PET tracers can be poor, and denoised images may have inherent biases affecting use with unknown data, such as disease, scanner, and noise levels, which are not included in the training data. To overcome these challenges, self-supervised and unsupervised learning approaches have attracted a steadily growing interest. Self-supervised learning generally refers to training algorithms that use self-labels automatically generated from unannotated data. Noise2Noise is a representative self-supervised denoising approach that restores clean images from multiple independent corrupt images [132] and is also reported to be effective for PET image denoising [133, 134]. To avoid the constraints of Noise2Noise, which requires more than one noise realization, Noise2Void, an unsupervised approach using a blind-spot network design [135], has also been used for PET image denoising [136].

**Fig. 5:**
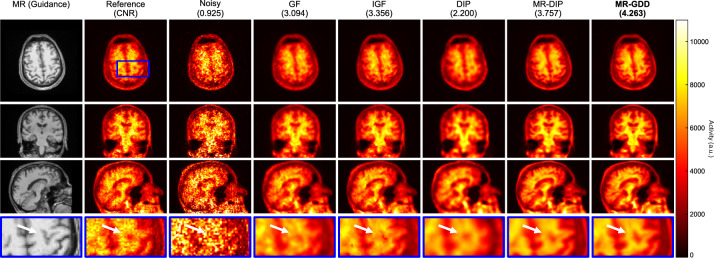
Examples of the denoised brain ^18^F-florbetapir PET images by unsupervised learning approaches. From left to right, the sample images showing the MR, standard-count PET, noisy PET, and denoised PET images corresponding to the Gaussian filter (GF), image-guided filter (IGF), DIP, MR-DIP (CDIP), and MR-guided deep decoder (GDD). © 2021 Elsevier. Reprinted with permission from Onishi et al. [143]

Among the unsupervised learning approaches, the deep image prior (DIP), which uses a CNN structure as an intrinsic regularizer and does not require the preparation of a prior training dataset [137], has achieved better performance in PET image denoising [138–140]. DIP training is formulated as follows:

16 $$\begin{array}{*{20}c} {\theta^{*} = \mathop {{\text{argmin}}}\limits_{\theta } \Vert {\varvec{x}}_{0} - f\left( {\theta {|}{\varvec{z}}} \right)\Vert, {\varvec{x}}^{*} = f\left( {\theta^{*} {|}{\varvec{z}}} \right),} \\ \end{array}$$

where $\Vert \bullet \Vert$ is the L2 loss, $f$ represents the CNN model with trainable parameters $\theta$, the training label ${{\varvec{x}}}_{0}$ is the noisy PET image, and ${\varvec{z}}$ is the network input. After reaching an optimal stopping criterion, the CNN outputs the final denoised PET image, ${{\varvec{x}}}^{*}$. Conditional DIP (CDIP) [141, 142], which uses anatomical information instead of the original random noise as the network input, promotes denoising performance, and an attention mechanism to weight the multi-scale features extracted from the anatomical image guides the spatial details and semantic features of the image more effectively (Fig. 5) [143]. A four-dimensional DIP can perform end-to-end dynamic PET image denoising by introducing a feature extractor and several dozen reconstruction branches [144]. Recently, a pre-trained model using population information from a large number of existing datasets has been shown to improve DIP-based PET image denoising [145]. Furthermore, the self-supervised pre-training model acquired transferable and generalizable visual representations from only low-quality PET images; it achieves robust denoising performance for various PET tracers and scanner data [146].

### Emerging approaches

Currently, deep learning-based PET image restoration technology has already been implemented in commercial PET scanners [13, 14, 147] with Food and Drug Administration-cleared commercially available software [148–152], making significant contributions in clinical practice. Moreover, deep learning continues to develop rapidly, with emerging approaches and novel applications being frequently proposed.

**Fig. 6:**
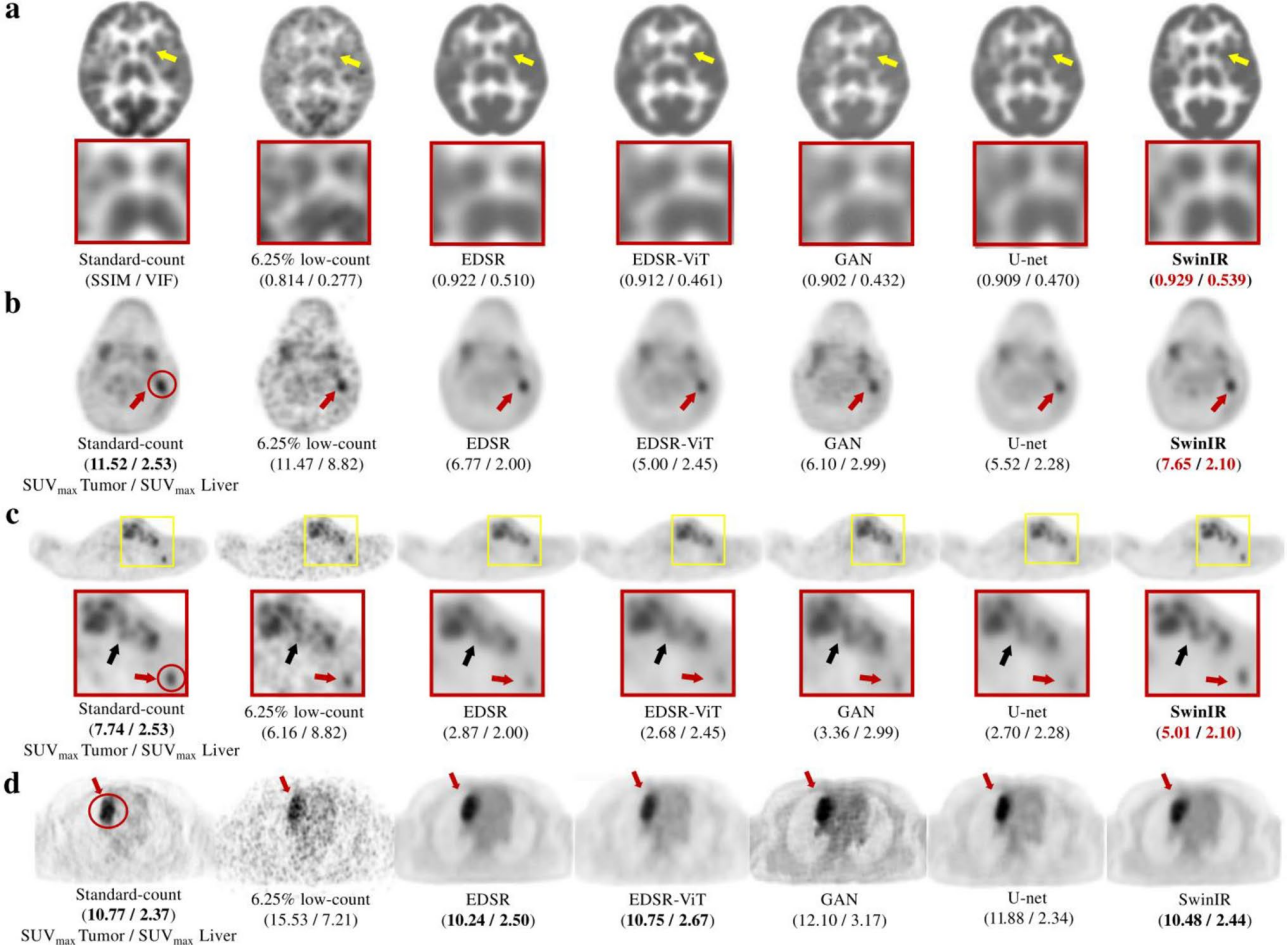
Examples of denoised ^18^F-FDG PET images by emerging approaches. Each column (a) to (d) indicates different patients or organs. From left to right, the sample images show the standard-count PET, low-count PET, and denoised PET images, corresponding to the enhanced deep super-resolution network (EDSR), EDSR-ViT, GAN, U-Net, and Swin Transformer image restoration network (SwinIR). © 2023 SNCSC. Reprinted with permission from Wang et al. [159]

The transformer architecture revolutionizes sequence tasks with self-attention and efficiently captures distant dependencies [153]. In particular, the Vision Transformer (ViT) [154] and Swin Transformer [155] effectively handle both local and global features, more so than CNNs. These transformer models have been adapted for PET image denoising and in some cases have reported to outperform CNN-based denoising performance (Fig. 6) [156–161]. The emergence of diffusion models resulted in a breakthrough in the field of image generation, following variational autoencoders and GANs. The effectiveness of denoising diffusion probabilistic models [162] for PET image denoising has also been investigated [163, 164]. From the viewpoint of personal information protection, federated learning, which enables decentralized learning without the need to export clinical data, is beginning to be applied to PET image denoising [165, 166]. In addition, uncertainty estimation [167, 168] and noise-aware networks [169–171] can provide additional value to conventional denoising methods. The advancement of PET state-of-the-art scanners, represented currently by total-body PET scanners [172], will pave the way for further applications of deep learning.

## Deep learning for direct PET image reconstruction

Deep learning-based direct PET image reconstruction is a data-driven approach in which the reconstructed PET image, ***x***, can be directly transformed from the measurement data, ***y***, through a neural-network model, *f*, with trainable weights, $\theta$. This is expressed as a problem of minimizing the following objective function:

17 $$\begin{array}{*{20}c} {\theta^{*} = \mathop {{\text{argmin}}}\limits_{\theta } E\left( {f\left( {\theta {|}{\varvec{y}}} \right);{\varvec{x}}} \right),} \\ \end{array}$$

where *E* is a loss function, such as the MSE. The direct reconstruction approach is completely different from previous approaches in that it attempts to find an image reconstruction mechanism entirely from the training dataset without involving physical models, such as forward or backprojection.

**Fig. 7:**
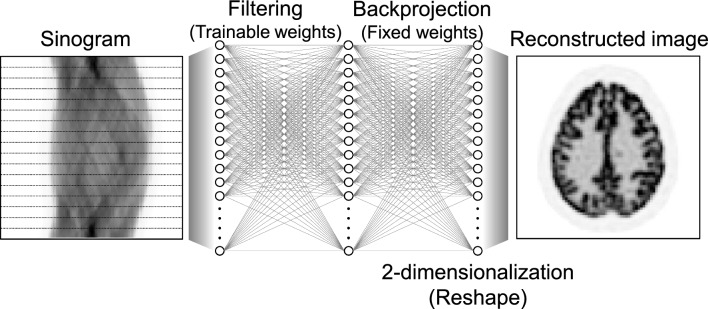
Schematic illustration of the earliest direct image reconstruction algorithm for SPECT by Floyd in 1991 [173]. The network realizes a data-driven FBP method in which the first hidden layer works as a trainable kernel in the projection data space, and the second hidden layer works as a backprojection. Note that the first hidden layer performed 1D filtering in the actual implementation using the common trainable weights at each angle

The earliest direct image reconstruction algorithm in the field of nuclear medicine was probably a single-photon emission CT (SPECT) image reconstruction algorithm using a perceptron with two hidden layers, as proposed by Floyd in 1991 [173], before the advent of deep learning. In this method, a four-layer perceptron was used, consisting of an input layer that considers the measurement sinogram as 1D data, one trainable hidden layer, another hidden layer with fixed weights for the backprojection calculation, and an output layer, as shown in Fig. 7. This pioneering work demonstrated that it was possible to realize a data-driven FBP method in which the first hidden layer works as a trainable kernel in the projection data space, and the second hidden layer works as a backprojection. The trained kernel in the projection data space delivers a kernel similar to that corresponding to a ramp filter in the frequency domain, as would be expected.

**Fig. 8:**
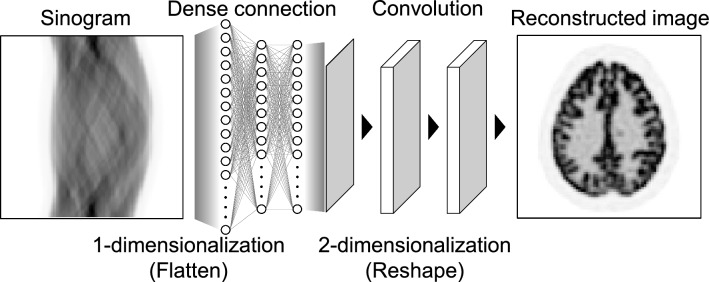
Schematic illustration of the AUTOMAP architecture by Zhou et al. in 2018 [174]. The network introduces dense connections in the first and second layers of the neural-network structure, which can work as an inverse transformation from measurement data to the reconstructed images in global operation

After 27 years, the advent of automated transform by manifold approximation (AUTOMAP) proposed by Zhou et al. in 2018 [174] led to the development of more modern direct image reconstruction algorithms using both fully connected (FC) layer as well as CNNs. The AUTOMAP architecture introduces dense (FC) connections in the first and second layers of the neural-network structure, as shown in Fig. 8. An interesting aspect of the dense connections in the AUTOMAP architecture is that they can work as an inverse transformation from measurement data to reconstructed MR and PET images in global operations using dense connections.

**Fig. 9:**
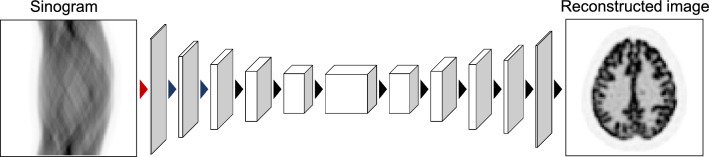
Schematic illustration of the DeepPET architecture by Häggström et al. in 2019 [175]. The arrows collectively represent the two convolution layers. The encoder part initially utilizes larger convolution filter kernel sizes of 7 × 7 in the red arrow and 5 × 5 in the blue arrows to work wider operation in the sinogram space, similar to the global operation with dense connections in the AUTOMAP architecture

Inspired by the success of AUTOMAP, Häggström et al. proposed the DeepPET method for direct PET image reconstruction using an FCN architecture [175], as shown in Fig. 9. The DeepPET architecture consists of an encoder–decoder network that mimics some modifications of the VGG16 network [176], which has several improvements to address the challenges of using FCNs for direct image reconstruction. First, the encoder part initially utilizes larger convolution filter kernel sizes to perform a wider operation in the sinogram space, similar to the global operation with dense connections in the AUTOMAP architecture. Second, a deeper layered network structure allows the bottleneck features to obtain better latent representations. The size of the bottleneck feature is 18 × 17 × 1024, indicating that almost no spatial information of the input sinogram remains. The DeepPET has much fewer trainable parameters than the AUTOMAP, which uses dense connection layers (approximately 800 million trainable parameters for AUTOMAP compared to approximately 60 million for DeepPET [18]) and can train with a smaller dataset. DPIR-Net, a network structure similar to DeepPET, improves PET image quality by adding perceptual and adversarial losses to the loss function [177]. In addition, direct image reconstruction for long-axial field-of-view PET scanners has been developed [178]. DirectPET, which incorporates a Radon inversion layer that connects a masked region of the sinogram to a local patch of the image in the neural network as a PET physical model, has also been proposed [179, 180]. Furthermore, direct image reconstruction using modern network structures, such as a transformer network, and physics-informed networks have been developed [181, 182].

**Fig. 10:**
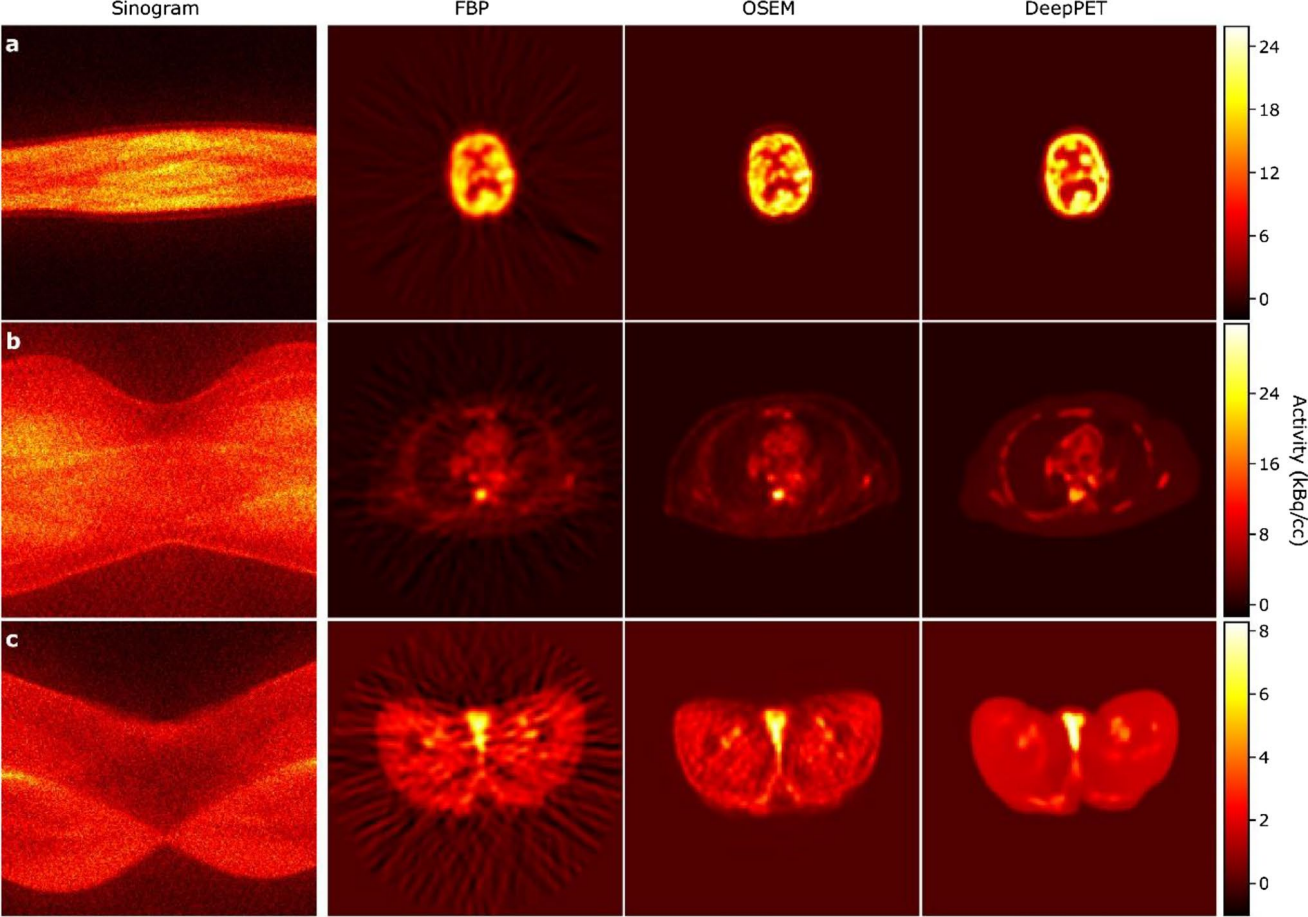
Input sinograms and the reconstructed results of DeepPET method [175]. Columns correspond to the input sinogram, FBP, OSEM, and DeepPET results, respectively (left to right). © 2019 Elsevier. Reprinted with permission from Häggström et al. [175]

These direct PET image reconstruction algorithms are expected to represent the next generation of fast and accurate image reconstruction methods; however, they have some limitations. We consider the DeepPET reconstruction results shown in Fig. 10 as an example. At a first glance, the direct reconstruction algorithms produce good PET images from sinograms. However, the detailed structures may differ from those obtained using the OSEM algorithm. This discrepancy may arise because obtaining an accurate inverse transformation from sinograms to reconstructed images using a data-driven approach is challenging. Consequently, these algorithms may generate artifacts or false structures in the reconstructed PET images. Another critical challenge is that the algorithms are limited to 2D image reconstruction, owing to graphics processing unit memory capacity. Therefore, these algorithms require a large number of training datasets to learn the backprojection task in a data-driven manner.

**Fig. 11:**
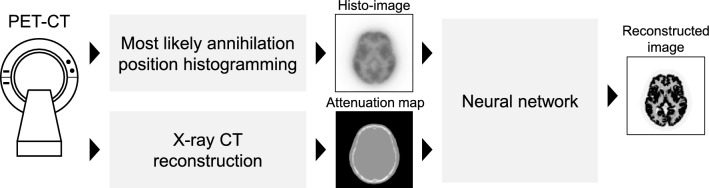
Schematic illustration of the FastPET framework for TOF-PET image reconstruction by Whiteley et al. in 2021 [184]

**Fig. 12:**
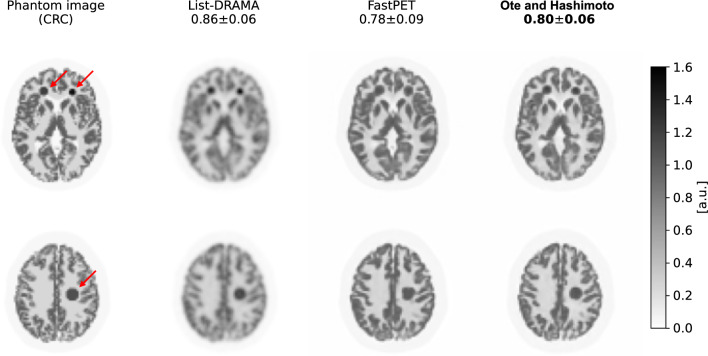
Results of FastPET reconstruction. Columns correspond to the phantom image, list-mode DRAMA, FastPET without and with direction information (from left to right). Reconstructed images were tagged using the mean and standard deviation of the contrast recovery coefficients (CRCs) of three tumor regions. The use of directional information (Ote and Hashimoto [186]) improves reconstruction performance (FastPET [184]). The figure is reprinted with a modification from the work of Ote and Hashimoto [186]

Another strategy for direct PET image reconstruction involves the use of TOF information. In general, acquired PET data (list-mode format data) are first histogrammed into the sinogram space. However, a different strategy directly creates histograms of the acquired PET data in the image space, known as the histo-image [183]. Whiteley et al. proposed the FastPET method, which obtains accurate PET images with a faster calculation time from the histo-image blurred by the TOF resolution in the LOR direction for each event (Fig. 11) [184]. The FastPET method differs from other direct image reconstruction methods, such as AUTOMAP and DeepPET, because the FastPET framework uses input images in the image space instead of the sinogram space. This implies that FastPET can employ CNNs, such as the U-Net structure. The advantage of this strategy is that it can be easily extended to 3D PET data because the sizes of the input data and network structure are quite small compared to those in other direct image reconstruction algorithms. Furthermore, some improved methods use the direction information of the acquired PET data by dividing the histo-image into several projection angles (Fig. 12) [185–187].

## Deep learning for iterative PET image reconstruction

Deep learning-based iterative PET image reconstruction is a hybrid approach that combines existing iterative PET image reconstruction algorithms based on physical and statistical models with deep learning algorithms. There are two main approaches: one involves incorporating a neural network as an equality constraint, and the other involves integrating a neural network into the objective function as a penalty. The former approach to synthetic PET image reconstruction is represented by the following equation:

$$\hat{\user2{x}} = \mathop {{\text{argmin}}}\limits_{{\varvec{x}}} L\left( {{\varvec{y}}{|}{\varvec{x}}} \right),$$

18 $$\begin{array}{*{20}c} {s.t. x = f\left( {\theta {|}{\varvec{z}}} \right),} \\ \end{array}$$

where *L* is the negative Poisson log-likelihood function and ***z*** is the input to the neural network, *f*, with trainable weights, $\theta$. A simpler solution involves utilizing the pre-trained model *f* for the PET image denoising task and updating the reconstructed PET image.^1^ This optimization problem is solved such that the measurement data align with the projection of the denoised PET image output from the neural network. In other words, the denoised PET images from the neural network were as consistent with the measurement data as possible, although they were the output of the neural-network denoising.

**Fig. 13:**
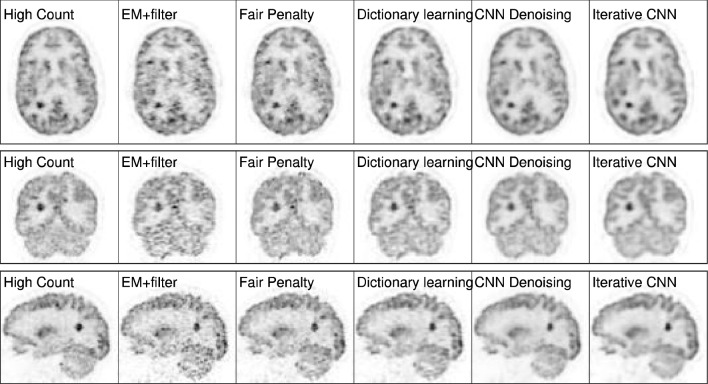
Reconstructed results of the iterative PET image reconstruction algorithm using CNN representation [188]. Columns represent high count ground truth, EM reconstruction with the Gaussian filtering, fair penalty-based penalized reconstruction, dictionary learning-based reconstruction [198], CNN denoising, and the proposed iterative PET image reconstruction using CNN. © 2019 IEEE. Reprinted with permission from Gong et al. [188]

**Fig. 14:**
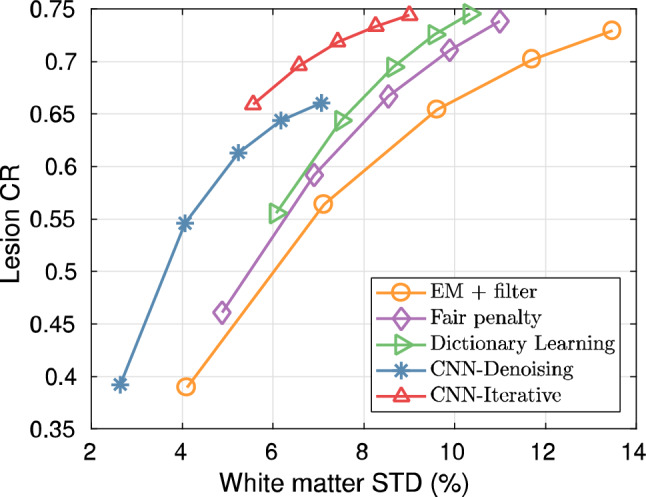
Tradeoffs of the iterative PET image reconstruction algorithm using CNN representation [188] between the lesion contrast recovery (CR) and standard deviation (STD) of the white matter region. Legends represent the same methods as in Fig. 13. © 2019 IEEE. Reprinted with permission from Gong et al. [188]

**Fig. 15:**
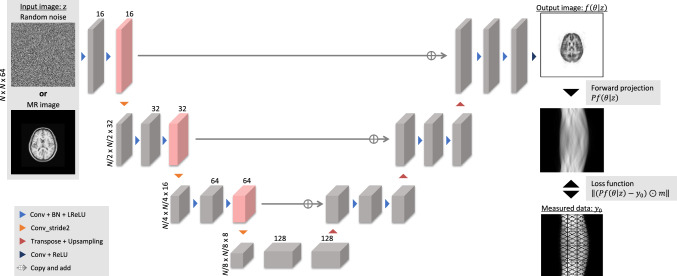
Overview of the iterative PET image reconstruction using the DIP framework [195]. This is a simple image reconstruction method incorporating a forward projection model as a loss function by backpropagation. © 2022 IEEE. Reprinted with permission from Hashimoto et al. [195]

**Fig. 16:**
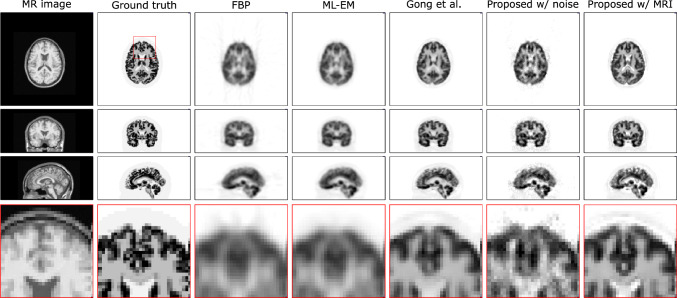
Reconstructed results of the iterative PET image reconstruction using the DIP framework [195]. Columns represent MR image, ground truth, FBP, MLEM with Gaussian filtering, DIP reconstruction by Gong et al. [192], proposed methods with random noise and MRI input [195].The proposed method with MRI input is visually close to the ground truth. © 2022 IEEE. Reprinted with permission from Hashimoto et al. [195]

Gong et al. proposed an iterative PET image reconstruction algorithm using a synthesis-based prior [188]. The algorithm transforms the constrained optimization problem in Eq. (17) into an unconstrained optimization problem using the augmented Lagrangian format and is solved using the alternating direction method of multipliers (ADMM) algorithm [189]. The reconstructed results of the method by Gong et al. achieved superior performance in terms of lesion contrast and white matter noise tradeoff, as shown in Figs. 13 and 14, respectively. This framework can be reasonably extended by modifying the denoiser network. For example, Xie et al. replaced the network with a GAN generator incorporating a self-attention mechanism [190] to enhance the image quality without introducing blurring [191]. This method produced a better lesion contrast recovery and background noise tradeoff than the other methods. Alternatively, high-quality PET images can be obtained without any prior training dataset by introducing a DIP as a constraint, which uses the intrinsic prior of the CNN structure [192]. Ote et al. implemented 3D list-mode PET image reconstruction using DIP [193] by replacing the negative log-likelihood function in Eq. (17) with a list-mode log-likelihood function [194]. Additionally, some iterative reconstruction methods have also been proposed using only backpropagation without any backprojection process (Figs. 15 and 16) [195–197].

Next, we considered the latter approach for iterative PET image reconstruction using an analysis-based prior as follows:

19 $$\begin{array}{*{20}c} {\hat{\user2{x}} = \mathop {{\text{argmin}}}\limits_{{\varvec{x}}} L\left( {{\varvec{y}}{|}{\varvec{x}}} \right) + \beta R\left( {\varvec{x}} \right),} \\ \end{array}$$

where *R* is an energy function, modulated the influence by the regularization parameter, *β*. For example, we consider a simpler case, where the energy function is as follows:

20 $$\begin{array}{*{20}c} {R\left( {\varvec{x}} \right) = \mathop \sum \limits_{j = 1}^{J} \left( {f\left( {\theta {|}{\varvec{z}}} \right)_{j} - x_{j} } \right)^{2} ,} \\ \end{array}$$

where j denotes the voxel index. Intuitively, Eq. (19) is less constraining than the synthetic PET image reconstruction because optimization is performed to ensure that the reconstructed PET image does not deviate far from the neural-network output in the image space [18], while not requiring equality with the output of the neural network.

**Fig. 17:**
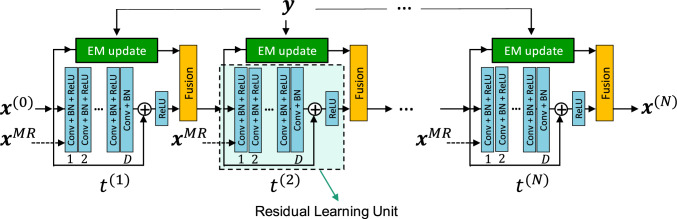
Overview of the FBSEM-Net [199]. The method can control the regularization parameter in the fusion block as the trainable weight. © 2021 IEEE. Reprinted with permission from Mehranian and Reader [199]

**Fig. 18:**
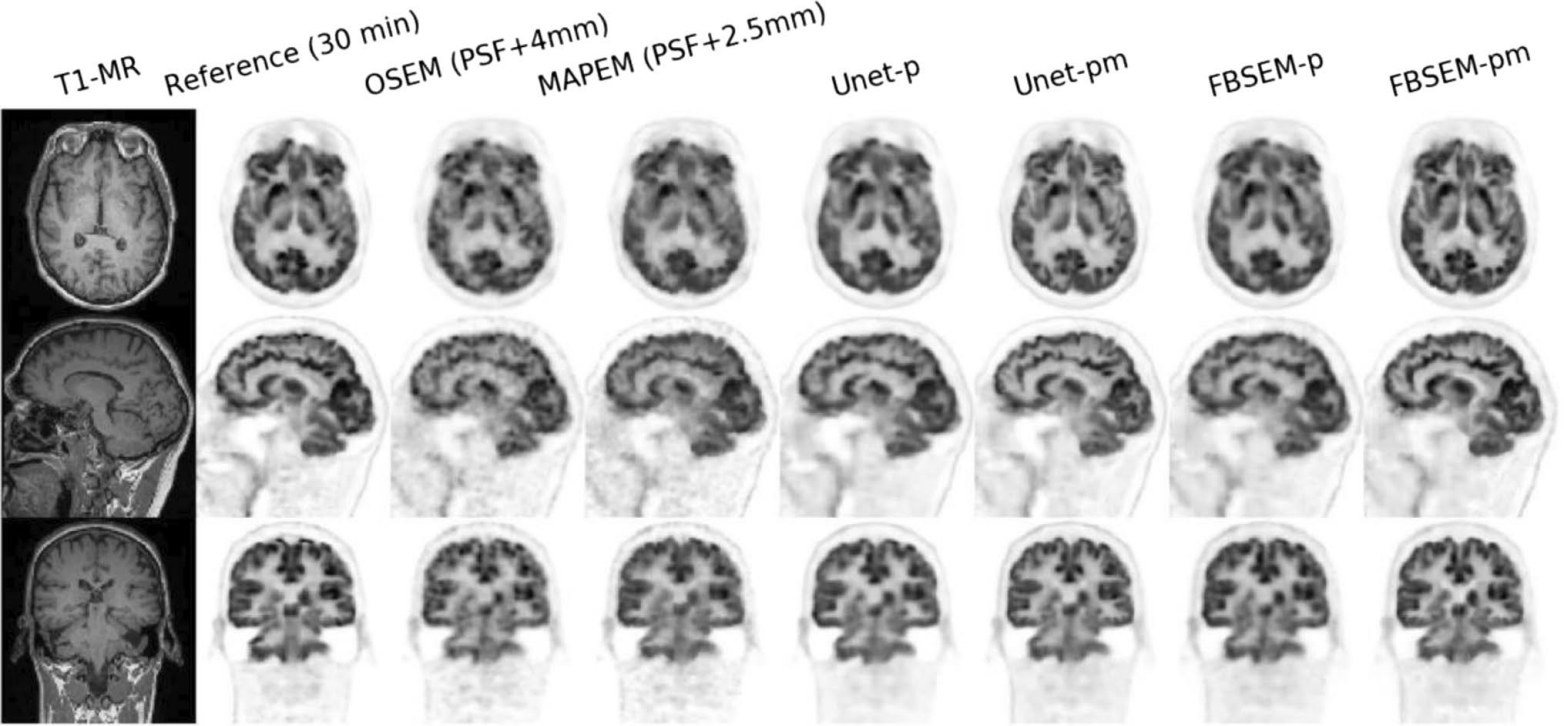
Reconstructed results of the FBSEM-Net [199]. The -p and -pm in the methods represent the use of PET and MRI data for input, respectively. © 2021 IEEE. Reprinted with permission from Mehranian and Reader [199]

Mehranian and Reader proposed PET image reconstruction via FBSEM-Net [199], which uses a forward–backward splitting algorithm [200]. The FBSEM-Net architecture is illustrated in Fig. 17. Using PET-MR data, FBSEM-Net can enhance PET image quality compared to other conventional reconstruction algorithms, as shown in Fig. 18. Kim et al. proposed a deep learning-based iterative PET image reconstruction [201] that introduced local linear fitting inspired by guided filtering [202] to the energy function for bias reduction in blind denoising, which is as follows:

21 $$\begin{array}{*{20}c} {R\left( {\varvec{x}} \right) = \frac{1}{2}\Vert{\varvec{x}} - q \odot f\left( {\theta {|}{\varvec{x}}} \right) - b\Vert_{2}^{2} ,} \\ \end{array}$$

where ***x*** is the PET image, *f* is the denoiser network with weights, $\theta$, ⊙ is the Hadamard product, and *q* and *b* denote the local linear fitting coefficients. The method was divided into substeps for the denoiser network and local linear fitting using the ADMM algorithm. Gong et al. proposed MAPEM-Net, which can be easily implemented by incorporating a potential function into neural-network optimization [203]. In addition, various other iterative PET image reconstruction algorithms have been proposed for PET and SPECT [204–216].

## Deep learning for dynamic PET image reconstruction

PET can be used to analyze the temporal pharmacokinetics of PET tracers through continuous measurements after the administration of radiopharmaceuticals. Usually, kinetic parameters, such as Ki, are estimated by fitting compartment models to the dynamic PET images of each voxel reconstructed over short-time frames. Alternatively, direct parametric reconstruction algorithms for dynamic PET data have been developed to enable accurate noise modeling [217–219].

With the advancement of deep learning, several dynamic PET image reconstruction methods using CNN have been proposed [220–223]. Li et al. expanded the DeepPET algorithm to direct the parametric image reconstruction from small-frame sinograms without using an input function [224]. Gong et al. introduced direct linear parametric PET image reconstruction using a nonlocal DIP architecture [225] with a linear kinetic model layer [226].

Dual-tracer PET imaging can measure two PET tracers in a single scan, which may be useful for diagnosing and tracking diseases as another application of dynamic PET [227, 228]. Deep learning has been reported to be useful for these approaches [229–234].

## Conclusion and future perspectives

We conducted a comprehensive review of deep learning-based PET image denoising and reconstruction. Remarkable strides in deep learning-based PET image reconstruction are noteworthy. Recent advancements in PET scanner innovations are equally impressive and have aligned seamlessly with progress made in the field of deep learning technology. One of the recent breakthroughs in PET hardware is total-body PET geometry [235–237] that obtains high-sensitivity PET data and can provide extremely less noisy training datasets for deep learning-based PET image reconstruction [238]. Another noteworthy innovation is the TOF technology discussed in Sect. 4. Along with advancements in PET detectors [239–242], ultrafast TOF detectors of 30 ps have been developed, enabling reconstruction-free positron emission imaging [243]. This synergy between state-of-the-art TOF and deep learning technologies has pushed the limits of TOF performance [244–246]. Undoubtedly, the integration of deep learning will play a pivotal role in enhancing the performance of not only PET imaging but also signal processing [247–250].

## Footnotes

1 The updated image for each iteration can be used for neural-network input ***z***.

## References

[1] LeCun Y, Bengio Y, Hinton G (2015). Deep learning. Nature.

[2] Goodfellow IJ, Bengio Y, Courville A. Deep learning. Cambridge, MA, USA: MIT Press; 2016 http://www.deeplearningbook.org.

[3] Schmidhuber J (2015). Deep learning in neural networks: An overview. Neural Netw.

[4] Suzuki K (2017). Overview of deep learning in medical imaging. Radiol Phys Technol.

[5] Litjens G, Kooi T, Bejnordi BE, Setio AAA, Ciompi F (2017). A survey on deep learning in medical image analysis. Med Image Anal.

[6] Shen D, Wu G, Suk H (2017). Deep learning in medical image analysis. Annu Rev Biomed Eng.

[7] Lee JG, Jun S, Cho YW, Lee H, Kim GB (2017). Deep learning in medical imaging: general overview. Korean J Radiol.

[8] Fujita H (2020). AI-based computer-aided diagnosis (AI-CAD): the latest review to read first. Radiol Phys Technol.

[9] Kaji S, Kida S (2019). Overview of image-to-image translation by use of deep neural networks: denoising, super-resolution, modality conversion, and reconstruction in medical imaging. Radiol Phys Technol.

[10] Matsubara K, Ibaraki M, Nemoto M, Watabe H, Kimura Y (2022). A review on AI in PET imaging. Ann Nucl Med.

[11] PET with Advanced Intelligent Clear IQ-Engine | Nuclear Medicine | Canon Medical Systems. https://global.medical.canon/products/nuclear_medicine/PET-with-Advanced. Accessed 15 August 2023.

[12] FUJIFILM Endoscopy. https://www.fujifilm-endoscopy.com/cadeye. Accessed 15 August 2023.

[13] Wang T, Qiao W, Wang Y, Wang J, Lv Y (2022). Deep progressive learning achieves whole-body low-dose ^18^F-FDG PET imaging. EJNMMI Phys.

[14] Mehranian A, Wollenweber SD, Walker MD, Bradley KM, Fielding PA (2022). Deep learning–based time-of-flight (ToF) image enhancement of non-ToF PET scans. Eur J Nucl Med Mol Imaging.

[15] Liu F, Jang H, Kijowski R, Bradshaw T, McMillan AB (2018). Deep learning MR imaging–based attenuation correction for PET/MR imaging. Radiology.

[16] Liu F, Jang H, Kijowski R, Zhao G, Bradshaw T, McMillan AB (2018). A deep learning approach for ^18^F-FDG PET attenuation correction. EJNMMI Phys.

[17] Gong K, Berg E, Cherry SR, Qi J (2019). Machine learning in PET: from photon detection to quantitative image reconstruction. Proc IEEE.

[18] Reader AJ, Corda G, Mehranian A, da Costa-Luis C, Ellis S, Schnabel JA (2020). Deep learning for PET image reconstruction. IEEE Trans Radiat Plasma Med Sci.

[19] Lee JS (2020). A review of deep-learning-based approaches for attenuation correction in positron emission tomography. IEEE Trans Radiat Plasma Med Sci.

[20] Hashimoto F, Ito M, Ote K, Isobe T, Okada H, Ouchi Y (2021). Deep learning-based attenuation correction for brain PET with various radiotracers. Ann Nucl Med.

[21] Gong K, Kim K, Cui J, Wu D, Li Q (2021). The evolution of image reconstruction in PET: From filtered back-projection to artificial intelligence. PET Clin.

[22] Liu J, Malekzadeh M, Mirian N, Song TA, Liu C, Dutta J (2021). Artificial intelligence-based image enhancement in PET imaging: Noise reduction and resolution enhancement. PET Clin.

[23] Reader AJ, Schramm G (2021). Artificial Intelligence for PET Image Reconstruction. J Nucl Med.

[24] Reader AJ, Pan B (2023). AI for PET image reconstruction. Br J Radiol.

[25] Phelps ME (2012). PET: molecular imaging and its biological applications.

[26] Schoder H, Gonen M (2007). Screening for cancer with PET and PET/CT: potential and limitations. J Nucl Med.

[27] Minamimoto R, Senda M, Uno K, Jinnouchi S, Iinuma T (2007). Performance profile of FDG-PET and PET/CT for cancer screening on the basis of a Japanese Nationwide Survey. Ann Nucl Med.

[28] Zhu L, Ploessl K, Kung HF (2014). PET/SPECT imaging agents for neurodegenerative diseases. Chem Soc Rev.

[29] Barthel H, Schroeter ML, Hoffmann KT, Sabri O (2015). PET/MR in dementia and other neurodegenerative diseases. Semin Nucl Med.

[30] Jones T, Rabiner EA (2012). The development, past achievements, and future directions of brain PET. J Cereb Blood Flow Metab.

[31] Onishi Y, Isobe T, Ito M, Hashimoto F, Omura T, Yoshikawa E (2022). Performance evaluation of dedicated brain PET scanner with motion correction system. Ann Nucl Med.

[32] National Research Council (2006) Health risks from exposure to low levels of ionizing radiation: BEIR VII phase 2. The National Academies Press, Washington, DC. 10.17226/11340

[33] Ramachandran GN, Lakshminarayanan AV (1971). Three-dimensional reconstruction from radiographs and electron micrographs: Application of convolutions instead of Fourier transforms. Proc Natl Acad Sci.

[34] Shepp LA, Logan BF (1974). The Fourier reconstruction of a head section. IEEE Trans Nucl Sci.

[35] Tanaka E, Iinuma T (1975). Correction functions for optimizing the reconstructed image in transverse section scan. Phys Med Biol.

[36] Defrise M, Kinahan PE (1998). Data acquisition and image reconstruction for 3D PET in The Theory and Practice of 3D PET.

[37] Radon J (1986). On the determination of functions from their integral values along certain manifolds. IEEE Trans Med Imaging.

[38] Colsher JG (1980). Fully three-dimensional positron emission tomography. Phys Med Biol.

[39] Kinahan PE, Rogers JG (1989). Analytic 3D image reconstruction using all detected events. IEEE Trans Nucl Sci.

[40] Townsend DW, Spinks TJ, Jones T, Geissbühler A, Defrise M (1989). Three-dimensional reconstruction of PET data from a multi-ring camera. IEEE Trans Nucl Sci.

[41] Townsend DW, Geissbühler A, Defrise M, Hoffman EJ, Spinks TJ (1991). Fully three-dimensional reconstruction for a PET camera with retractable septa. IEEE Trans Med Imag.

[42] Townsend DW, Bendriem B. Introduction to 3D PET. In: Bendriem B, Townsend DW, eds. The Theory and Practice of 3D PET. Dordrecht: Springer; 1998.

[43] Grootoonk S, Spinks TJ, Michel C, Jones T, Correction for scatter using a dual energy window technique in a tomograph operated without septa. In,  (1991). IEEE Medical Imaging Conference Record. Nuclear Science Symposium and Medical Imaging Conference.

[44] Bendriem B, Trébossen R, Frouin V, Scatter SAAPET, Acquisitions CUS, with Low and High Lower Energy Thresholds. In,  (1993). IEEE Medical Imaging Conference Record. Nuclear Science Symposium and Medical Imaging Conference.

[45] Bailey DL, Meikle SR (1994). A convolution-subtraction method for 3D PET. Phys Med Biol.

[46] Ollinger JM (1996). Model-Based Scatter Correction for Fully 3D PET. Phys Med Biol.

[47] Watson CC, Newport D, Casey ME. A single scatter simulation technique for scatter correction in 3D PET. In: Three-Dimensional Image Reconstruction in Radiology and Nuclear Medicine. Dordrecht, The Netherlands: Kluwer Acad.; 1996. p. 255–268.

[48] Thielemans K, Manjeshwar M, Tsoumpas C, Jansen FP. A new algorithm for scaling of PET scatter estimates using all coincidence events. In: 2007 IEEE IEEE Nuclear Science Symposium Conference Record, pp. 3586–3590, doi: 10.1109/NSSMIC.2007.4436900.

[49] Watson CC, Casey ME, Michel C, Bendriem B (2020). Advances in scatter correction for 3D PET/CT. IEEE Trans Radiat Plasma Med Sci.

[50] Defrise M, Clackdoyle R, Townsend DW (1995). Image reconstruction from truncated, two-dimensional, parallel projections. Inverse Probl.

[51] Defrise M, Kinahan PE, Michel C, Rogers JG, Townsend DW, Clackdoyle R (1997). Exact and approximate rebinning algorithms for 3-D PET data. IEEE Trans Med Imaging.

[52] Kinahan PE, et al. Fast iterative image reconstruction of 3D PET data. In: 1996 IEEE Nuclear Science Symposium. Conference Record. Anaheim, CA, USA; 1996. p. 1918–22 vol.3. doi: 10.1109/NSSMIC.1996.588009.

[53] Comtat C, Kinahan PE, Defrise M, Townsend DW, Michel C (1998). Simultaneous reconstruction of activity and attenuation in the presence of singles events. IEEE Trans Nucl Sci.

[54] Obi T, Matej S, Lewitt RM, Herman GT. 2.5D simultaneous multi-slice reconstruction by iterative algorithms from Fourier-rebinned PET data. IEEE Trans Med Imaging. 2000;19:474–484. 10.1109/42.870257 11021690

[55] Shepp LA, Varidi Y (1982). Maximum likelihood reconstruction for emission tomography. IEEE Trans Med Imaging.

[56] Lange K, Carson R (1984). EM reconstruction algorithm for emission and transmission tomography. J Comput Assist Tomogr.

[57] Vardi Y, Shepp LA, Kaufuman L (1985). A statistical model for positron emission tomography. J Amer Stat Assoc.

[58] Qi J, Leahy RM (2006). Iterative reconstruction techniques in emission computed tomography. Phys Med Biol.

[59] Hudson HM, Larkin RS (1994). Accelerated image reconstruction using ordered subsets of projection data. IEEE Trans Med Imaging.

[60] Browne J, de Pierro AB (1996). A row-action alternative to the EM algorithm for maximizing likelihood in emission tomography. IEEE Trans Med Imaging.

[61] Tanaka E, Kudo H (2003). Subset-dependent relaxation in block-iterative algorithm for image reconstruction in emission tomography. Phys Med Biol.

[62] Murayama H, Yamaya T. Eiichi Tanaka, Ph.D. (1927–2021): pioneer of the gamma camera and PET in nuclear medicine physics. Radiol Phys Technol. 2023;16:1–7. 10.1007/s12194-022-00693-z 36534344

[63] Qi J, Leahy RM, Cherry SR, Chatziioannou A, Farquhar TH (1998). High-resolution 3D Bayesian image reconstruction using the microPET small-animal scanner. Phys Med Biol.

[64] Reader AJ, Ally S, Bakatselos F, Manavaki R, Walledge RJ (2002). One-pass list-mode EM algorithm for high-resolution 3-D PET image reconstruction into large arrays. IEEE Trans Nucl Sci.

[65] Lee K, Kinahan PE, Fessler JA, Miyaoka RS, Janes M, Lewellen TK (2004). Pragmatic fully 3D image reconstruction for the MiCES mouse imaging PET scanner. Phys Med Biol.

[66] Yamaya T, Hagiwara N, Obi T, Yamaguchi M, Ohyama N (2005). Transaxial system models for jPET-D4 image reconstruction. Phys Med Biol.

[67] Panin VY, Kehren F, Michel C, Casey M (2006). Fully 3-D PET reconstruction with system matrix derived from point source measurements. IEEE Trans Med Imaging.

[68] Matej S, Lewitt RM (1995). Efficient 3D grids for image reconstruction using spherically-symmetric volume elements. IEEE Trans Nucl Sci.

[69] Reader AJ, Sureau FC, Comtat C, Trébossen R, Buvat I (2006). Joint estimation of dynamic PET images and temporal basis functions using fully 4D ML-EM. Phys Med Biol.

[70] Levitan E, Herman GT (1987). A maximum a posteriori probability expectation maximization algorithm for image reconstruction in emission tomography. IEEE Trans Med Imaging.

[71] Herbert T, Leachy R (1989). A generalized EM algorithm for 3-D Bayesian reconstruction from projection data using Gibbs priors. IEEE Trans Med Imaging.

[72] Green PJ (1990). Bayesian reconstructions from emission tomography data using a modified EM algorithm. IEEE Trans Med Imaging.

[73] De Pierro AR, Yamagishi MEB (2001). Fast EM-like methods for maximum “a posteriori” estimates in emission tomography. IEEE Trans Med Imaging.

[74] Nuyts J, Beque D, Dupont P, Mortelmans L (2002). A concave prior penalizing relative differences for maximum-a-posteriori reconstruction in emission tomography. IEEE Trans Nucl Sci.

[75] Alenius S, Ruotsalainen U (2002). Generalization of median root prior reconstruction. IEEE Trans Med Imaging.

[76] Snyder DL, Miller MI, Politte DG (1987). Noise and edge artifacts in maximum-likelihood reconstructions for emission tomography. IEEE Trans Med Imaging.

[77] Wilson DW, Tsui BMW, Barrett HH (1994). Noise properties of the EM algorithm: II. Monte Carlo simulations Phys Med Biol.

[78] Snyder DL, Miller MI (1985). The use of sieves to stabilize images produced with the EM algorithm for emission tomography. IEEE Trans Nucl Sci.

[79] Ahn S, Ross SG, Asma E, Miao J, Jin X (2015). Quantitative comparison of OSEM and penalized likelihood image reconstruction using relative difference penalties for clinical PET. Phys Med Biol.

[80] Comtat C, Kinahan PE, Fessler JA, Beyer T, Townsend DW (2002). Clinically feasible reconstruction of 3D whole-body PET/CT data using blurred anatomical labels. Phys Med Biol.

[81] Bowsher JE, Yuan H, Hedlund LW, Turkington TG, Akabani G, et al. Utilizing MRI information to estimate F18-FDG distributions in rat flank tumors. In: IEEE Nuclear Science Symposium and Medical Imaging Conference. 2004. pp. 2488–2492. doi: 10.1109/NSSMIC.2004.1462760.

[82] Nuyts J. The use of mutual information and joint entropy for anatomical priors in emission tomography. In: IEEE Nuclear Science Symposium and Medical Imaging Conference. 2007. pp. 4149–4154. doi: 10.1109/NSSMIC.2007.4437034.

[83] Mameuda Y, Kudo H. New anatomical-prior-based image reconstruction method for PET/SPECT. In: IEEE Nuclear Science Symposium and Medical Imaging Conference. 2007. pp. 4142–4148. doi: 10.1109/NSSMIC.2007.4437033.

[84] Bai B, Li Q, Leahy RM (2013). MR guided PET image reconstruction. Semin Nucl Med.

[85] Buades A, Coll B, Morel J-M. A non-local algorithm for image denoising. In: Proceedings of the IEEE Computer Society Conference on Computer Vision and Pattern Recognition. 2005;2 pp. 60–65. doi: 10.1109/CVPR.2005.38.

[86] Aharon M, Elad M, Bruckstein A (2006). K-SVD: An algorithm for designing overcomplete dictionaries for sparse representation. IEEE Trans Signal Process.

[87] Wang G, Qi J (2012). Penalized likelihood PET image reconstruction using patch-based edge-preserving regularization. IEEE Trans Med Imaging.

[88] Tang J, Yang B, Wang Y, Ying L (2016). Sparsity-constrained PET image reconstruction with learned dictionary. Phys Med Biol.

[89] Dong J, Kudo H (2016). Proposal of compressed sensing using nonlinear sparsifying transform for CT image reconstruction. Med Imag Tech.

[90] Xiang L, Qiao Y, Nie D, An L, Lin W (2017). Deep Auto-context Convolutional Neural Networks for Standard-Dose PET Image Estimation from Low-Dose PET/MRI. Neurocomputing.

[91] Ronneberger O, Fischer P, Brox T. U-Net: Convolutional Networks for Biomedical Image Segmentation. MICCAI 2015:234–241. doi: 10.1007/978-3-319-24574-4_28.

[92] Çiçek Ö, Abdulkadir A, Lienkamp SS, Brox T, Ronneberger O, Ourselin S, Joskowicz L, Sabuncu MR, Unal G, Wells W (2016). 3D U-Net: learning dense volumetric segmentation from sparse annotation. Medical Image Computing and Computer Assisted Intervention (MICCAI) LNCS.

[93] Sanaat A, Arabi H, Mainta I, Garibotto V, Zaidi H (2020). Projection Space Implementation of Deep Learning-Guided Low-Dose Brain PET Imaging Improves Performance over Implementation in Image Space. J Nucl Med.

[94] Schaefferkoetter J, Yan J, Ortega C, Sertic A, Lechtman E (2020). Convolutional neural networks for improving image quality with noisy PET data. EJNMMI Res.

[95] Liu H, Wu J, Lu W, Onofrey JA, Liu YH, Liu C (2020). Noise reduction with cross-tracer and cross-protocol deep transfer learning for low-dose PET. Phys Med Biol.

[96] Zhang K, Zuo W, Chen Y, Meng D, Zhang L (2017). Beyond a Gaussian Denoiser: Residual Learning of Deep CNN for Image Denoising. IEEE Trans Image Process.

[97] Liu CC, Huang HM (2019). Partial-ring PET image restoration using a deep learning based method. Phys Med Biol.

[98] Spuhler K, Serrano-Sosa M, Cattell R, DeLorenzo C, Huang C (2020). Full-count PET recovery from low-count image using a dilated convolutional neural network. Med Phys.

[99] Sano A, Nishio T, Masuda T, Karasawa K (2021). Denoising PET images for proton therapy using a residual U-net. Biomed Phys Eng Express.

[100] Mehranian A, Wollenweber SD, Walker MD, Bradley KM, Fielding PA (2022). Image enhancement of whole-body oncology [^18^F]-FDG PET scans using deep neural networks to reduce noise. Eur J Nucl Med Mol Imaging.

[101] Gong K, Guan J, Liu CC, Qi J (2019). PET Image Denoising Using a Deep Neural Network Through Fine Tuning. IEEE Trans Radiat Plasma Med Sci.

[102] Ladefoged CN, Hasbak P, Hornnes C, Højgaard L, Andersen FL (2021). Low-dose PET image noise reduction using deep learning: application to cardiac viability FDG imaging in patients with ischemic heart disease. Phys Med Biol.

[103] Xie Z, Li T, Zhang X, Qi W, Asma E, Qi J (2021). Anatomically aided PET image reconstruction using deep neural networks. Med Phys.

[104] Chen KT, Gong E, de Carvalho Macruz FB, Xu J, Boumis A (2019). Ultra-Low-Dose ^18^F-Florbetaben Amyloid PET Imaging Using Deep Learning with Multi-Contrast MRI Inputs. Radiology.

[105] Liu CC, Qi J (2019). Higher SNR PET image prediction using a deep learning model and MRI image. Phys Med Biol.

[106] Wang YR, Baratto L, Hawk KE, Theruvath AJ, Pribnow A (2021). Artificial intelligence enables whole-body positron emission tomography scans with minimal radiation exposure. Eur J Nucl Med Mol Imaging.

[107] Schramm G, Rigie D, Vahle T, Rezaei A, Van Laere K (2021). Approximating anatomically-guided PET reconstruction in image space using a convolutional neural network. Neuroimage.

[108] He Y, Cao S, Zhang H, Sun H, Wang F (2021). Dynamic PET Image Denoising With Deep Learning-Based Joint Filtering. IEEE Access.

[109] da Costa-Luis CO, Reader AJ (2021). Micro-Networks for Robust MR-Guided Low Count PET Imaging. IEEE Trans Radiat Plasma Med Sci.

[110] Chen KT, Schürer M, Ouyang J, Koran MEI, Davidzon G (2020). Generalization of deep learning models for ultra-low-count amyloid PET/MRI using transfer learning. Eur J Nucl Med Mol Imaging.

[111] Chen KT, Toueg TN, Koran MEI, Davidzon G, Zeineh M (2021). True ultra-low-dose amyloid PET/MRI enhanced with deep learning for clinical interpretation. Eur J Nucl Med Mol Imaging.

[112] Sun H, Jiang Y, Yuan J, Wang H, Liang D (2022). High-quality PET image synthesis from ultra-low-dose PET/MRI using bi-task deep learning. Quant Imaging Med Surg.

[113] Goodfellow I, Pouget-Abadie J, Mirza M, Xu B, Warde-Farley D, et al. Generative Adversarial Nets. In: Advances in neural information processing systems, vol. 27. 2014.

[114] Wang Y, Yu B, Wang L, Zu C, Lalush DS (2018). 3D conditional generative adversarial networks for high-quality PET image estimation at low dose. Neuroimage.

[115] Xue S, Guo R, Bohn KP, Matzke J, Viscione M (2022). A cross-scanner and cross-tracer deep learning method for the recovery of standard-dose imaging quality from low-dose PET. Eur J Nucl Med Mol Imaging.

[116] Hu Y, Lv D, Jian S, Lang L, Cui C (2023). Comparative study of the quantitative accuracy of oncological PET imaging based on deep learning methods. Quant Imaging Med Surg.

[117] Mirza M, Osindero S. Conditional Generative Adversarial Nets. arXiv preprint arXiv:1411.1784. 2014.

[118] Isola P, Zhu JY, Zhou T, Efros AA. Image-to-Image Translation with Conditional Adversarial Networks. arXiv preprint arXiv:1611.07004. 2018.

[119] Wang Y, Zhou L, Yu B, Wang L, Zu C (2019). 3D auto-context-based locality adaptive multi-modality GANs for PET synthesis. IEEE Trans Med Imaging.

[120] Fu Y, Dong S, Niu M, Xue L, Guo H (2023). AIGAN: Attention-encoding Integrated Generative Adversarial Network for the reconstruction of low-dose CT and low-dose PET images. Med Image Anal.

[121] Lu W, Onofrey JA, Lu Y, Shi L, Ma T (2019). An investigation of quantitative accuracy for deep learning based denoising in oncological PET. Phys Med Biol.

[122] Xue H, Teng Y, Tie C, Wan Q, Wu J (2020). A 3D attention residual encoder–decoder least-square GAN for low-count PET denoising. Nucl Instrum Methods Phys Res A.

[123] Ouyang J, Chen KT, Gong E, Pauly J, Zaharchuk G (2019). Ultra-low-dose PET reconstruction using generative adversarial network with feature matching and task-specific perceptual loss. Med Phys.

[124] Jeong YJ, Park HS, Jeong JE, Yoon HJ, Jeon K (2021). Restoration of amyloid PET images obtained with short-time data using a generative adversarial networks framework. Sci Rep.

[125] Gong Y, Shan H, Teng Y, Tu N, Li M (2021). Parameter-Transferred Wasserstein Generative Adversarial Network (PT-WGAN) for Low-Dose PET Image Denoising. IEEE Trans Radiat Plasma Med Sci.

[126] Zhu JY, Park T, Isola P, Efros AA. Unpaired Image-to-Image Translation using Cycle-Consistent Adversarial Networks. arXiv preprint arXiv:1703.10593. 2017.

[127] Lei Y, Dong X, Wang T, Higgins K, Liu T (2019). Whole-body PET estimation from low count statistics using cycle-consistent generative adversarial networks. Phys Med Biol.

[128] Zhou L, Schaefferkoetter JD, Tham IW, Huang G, Yan J (2020). Supervised learning with cyclegan for low-dose FDG PET image denoising. Med Image Anal.

[129] Zhao K, Zhou L, Gao S, Wang X, Wang Y (2020). Study of low-dose PET image recovery using supervised learning with CycleGAN. PLoS ONE.

[130] Sanaat A, Shiri I, Arabi H, Mainta I, Nkoulou R, Zaidi H (2021). Deep learning-assisted ultra-fast/low-dose whole-body PET/CT imaging. Eur J Nucl Med Mol Imaging.

[131] Ghafari A, Sheikhzadeh P, Seyyedi N, Abbasi M, Farzenefar S (2022). Generation of ^18^F-FDG PET standard scan images from short scans using cycle-consistent generative adversarial network. Phys Med Biol.

[132] Lehtinen J, Munkberg J, Hasselgren J, Laine S, Karras T, et al. Noise2Noise: Learning Image Restoration without Clean Data. arXiv preprint arXiv:1803.04189. 2018.

[133] Yie SY, Kang SK, Hwang D, Lee JS (2020). Self-supervised PET Denoising. Nucl Med Mol Imaging.

[134] Kang SK, Yie SY, Lee JS (2021). Noise2Noise Improved by Trainable Wavelet Coefficients for PET Denoising. Electronics.

[135] Krull A, Buchholz TO, Jug F. Noise2Void-Learning Denoising from Single Noisy Images. In: Proceedings of the IEEE/CVF Conference on Computer Vision and Pattern Recognition. 2019; pp. 2129–2137. doi: 10.1109/CVPR.2019.00223.

[136] Song TA, Yang F, Dutta J (2021). Noise2Void: unsupervised denoising of PET images. Phys Med Biol.

[137] Ulyanov D, Vedaldi A, Lempitsky V (2020). Deep image prior. Int J Comput Vis.

[138] Hashimoto F, Ohba H, Ote K, Teramoto A, Tsukada H (2019). Dynamic PET Image Denoising Using Deep Convolutional Neural Networks Without Prior Training Datasets. IEEE Access.

[139] Sun H, Peng L, Zhang H, He Y, Cao S, Lu L (2021). Dynamic PET Image Denoising Using Deep Image Prior Combined With Regularization by Denoising. IEEE Access.

[140] Yang CH, Huang HM (2022). Simultaneous Denoising of Dynamic PET Images Based on Deep Image Prior. J Digit Imaging.

[141] Cui J, Gong K, Guo N, Wu C, Meng X (2019). PET image denoising using unsupervised deep learning. Eur J Nucl Med Mol Imaging.

[142] Hashimoto F, Ohba H, Ote K, Teramoto A (2020). Unsupervised dynamic PET image denoising with anatomical information. Med Imaging Inf Sci.

[143] Onishi Y, Hashimoto F, Ote K, Ohba H, Ota R (2021). Anatomical-guided attention enhances unsupervised PET image denoising performance. Med Image Anal.

[144] Hashimoto F, Ohba H, Ote K, Kakimoto A, Tsukada H, Ouchi Y (2021). 4D deep image prior: dynamic PET image denoising using an unsupervised four-dimensional branch convolutional neural network. Phys Med Biol.

[145] Cui J, Gong K, Guo N, Wu C, Kim K (2021). Populational and individual information based PET image denoising using conditional unsupervised learning. Phys Med Biol.

[146] Onishi Y, Hashimoto F, Ote K, Matsubara K, Ibaraki M (2023). Self-Supervised Pre-Training for Deep Image Prior-Based Robust PET Image Denoising. IEEE Trans Radiat Plasma Med Sci.

[147] Tsuchiya J, Yokoyama K, Yamagiwa K, Watanabe R, Kimura K (2021). Deep learning-based image quality improvement of ^18^F-fluorodeoxyglucose positron emission tomography: a retrospective observational study. EJNMMI Phys.

[148] Chaudhari AS, Mittra E, Davidzon GA, Gulaka P, Gandhi H (2021). Low-count whole-body PET with deep learning in a multicenter and externally validated study. NPJ Digit Med.

[149] Katsari K, Penna D, Arena V, Polverari G, Ianniello A (2021). Artificial intelligence for reduced dose 18F-FDG PET examinations: a real-world deployment through a standardized framework and business case assessment. EJNMMI Phys.

[150] Weyts K, Lasnon C, Ciappuccini R, Lequesne J, Corroyer-Dulmont A (2022). Artificial intelligence-based PET denoising could allow a two-fold reduction in [^18^F]FDG PET acquisition time in digital PET/CT. Eur J Nucl Med Mol Imaging.

[151] Weyts K, Quak E, Licaj I, Ciappuccini R, Lasnon C (2023). Deep Learning Denoising Improves and Homogenizes Patient [^18^F]FDG PET Image Quality in Digital PET/CT. Diagnosis.

[152] Margail C, Merlin C, Billoux T, Wallaert M, Otman H (2023). Imaging quality of an artificial intelligence denoising algorithm: validation in ^68^Ga PSMA-11 PET for patients with biochemical recurrence of prostate cancer. EJNMMI Res.

[153] Vaswani A, Shazeer N, Parmar N, Uszkoreit J, Jones L, et al. Attention Is All You Need. arXiv preprint arXiv:1706.03762. 2017.

[154] Dosovitskiy A, Beyer L, Kolesnikov A, Weissenborn D, Zhai X, et al. An image is worth 16×16 words: Transformers for image recognition at scale. arXiv preprint arXiv:2010.11929. 2020.

[155] Liu Z, Lin Y, Cao Y, Hu H, Wei Y, et al. Swin Transformer: Hierarchical Vision Transformer using Shifted Windows. arXiv preprint arXiv:2103.14030. 2021.

[156] Luo Y, Wang Y, Zu C, Zhan B, Wu X, et al. 3D Transformer-GAN for High-Quality PET Reconstruction. In: Proceedings of the Medical Image Computing and Computer Assisted Intervention (MICCAI) 2021: 24th International Conference, Strasbourg, France, 2021, Part VI; 2021. pp. 276–285. doi: 10.1007/978-3-030-87231-1_27.

[157] Zhang L, Xiao Z, Zhou C, Yuan J, He Q (2022). Spatial adaptive and transformer fusion network (STFNet) for low-count PET blind denoising with MRI. Med Phys.

[158] Jang SI, Pan T, Li Y, Heidari P, Chen J (2023). Spach Transformer: Spatial and Channel-wise Transformer Based on Local and Global Self-attentions for PET Image Denoising. IEEE Trans Med Imaging.

[159] Wang YR, Wang P, Adams LC, Sheybani ND, Qu L (2023). Low-count whole-body PET/MRI restoration: an evaluation of dose reduction spectrum and five state-of-the-art artificial intelligence models. Eur J Nucl Med Mol Imaging.

[160] Wang YR, Qu L, Sheybani ND, Luo X, Wang J (2023). AI Transformers for Radiation Dose Reduction in Serial Whole-Body PET Scans. Radiol Artif Intell.

[161] Kruzhilov I, Kudin S, Vetoshkin L, Sokolova E, Kokh V. Whole-body PET image denoising for reduced acquisition time. arXiv preprint arXiv:2303.16085. 2023. 10.3389/fmed.2024.1415058 PMC11471667 39403284

[162] Ho J, Jain A, Abbeel P. Denoising Diffusion Probabilistic Models. arXiv preprint arXiv:2006.11239. 2020.

[163] Gong K, Johnson KA, Fakhri GE, Li Q, Pan T (2023). PET image denoising based on denoising diffusion probabilistic models. Eur J Nucl Med Mol Imaging.

[164] Han Z, Wang Y, Zhou L, Wang P, Yan B, et al. Contrastive Diffusion Model with Auxiliary Guidance for Coarse-to-Fine PET Reconstruction. arXiv preprint arXiv:2308.10157. 2023.

[165] Zhou B, Miao T, Mirian N, Chen X, Xie H (2023). Federated Transfer Learning for Low-Dose PET Denoising: A Pilot Study With Simulated Heterogeneous Data. IEEE Trans Radiat Plasma Med Sci.

[166] Zhou B, Xie H, Liu Q, Chen X, Guo X, et al. FedFTN: Personalized Federated Learning with Deep Feature Transformation Network for Multi-institutional Low-count PET Denoising. arXiv preprint arXiv:2304.00570. 2023. 10.1016/j.media.2023.102993 PMC10611438 37827110

[167] Sudarshan VP, Upadhyay U, Egan GF, Chen Z, Awate SP (2021). Towards lower-dose PET using physics-based uncertainty-aware multimodal learning with robustness to out-of-distribution data. Med Image Anal.

[168] Cui J, Xie Y, Joshi AA, Gong K, Kim K, et al. PET Denoising and Uncertainty Estimation Based on NVAE Model Using Quantile Regression Loss. In: International Conference on Medical Image Computing and Computer-Assisted Intervention. Cham: Springer Nature Switzerland; 2022. doi: 10.1007/978-3-031-16440-8_17

[169] Li Y, Cui J, Chen J, Zeng G, Wollenweber S, et al. A Noise-level-aware Framework for PET Image Denoising. arXiv preprint arXiv:2203.08034. 2022.

[170] Sanaei B, Faghihi R, Arabi H (2023). Employing Multiple Low-Dose PET Images (at Different Dose Levels) as Prior Knowledge to Predict Standard-Dose PET Images. J Digit Imaging.

[171] Xie H, Liu Q, Zhou B, Chen X, Guo X, Wang H, Li B, Rominger A, Shi K, Liu C (2023). Unified Noise-Aware Network for Low-Count PET Denoising With Varying Count Levels. IEEE Trans Radiat Plasma Med Sci.

[172] Zhang J, Cui Z, Jiang C, Guo S, Gao F, Shen D (2023). Hierarchical Organ-Aware Total-Body Standard-Dose PET Reconstruction From Low-Dose PET and CT Images. IEEE Trans Neural Netw Learn Syst.

[173] Floyd CE (1991). An artificial neural network for SPECT image reconstruction. IEEE Trans Med Imaging.

[174] Zhu B, Liu JZ, Cauley SF, Rosen BR, Rosen MS (2018). Image reconstruction by domain-transform manifold learning. Nature.

[175] Häggström I, Schmidtlein CR, Campanella G, Fuchs TJ (2019). DeepPET: A deep encoder–decoder network for directly solving the PET image reconstruction inverse problem. Med Image Anal.

[176] Simonyan K, Zisserman A. Very deep convolutional networks for large-scale image recognition. arXiv preprint arXiv:1409.1556.

[177] Hu Z, Xue H, Zhang Q, Gao J, Zhang N (2020). DPIR-Net: Direct PET image reconstruction based on the Wasserstein generative adversarial network. IEEE Trans Radiat Plasma Med Sci.

[178] Ma R, Hu J, Sari H, Xue S, Mingels C (2022). An encoder-decoder network for direct image reconstruction on sinograms of a long axial field of view PET. Eur J Nucl Med Mol Imaging.

[179] Whiteley W, Luk WK, Gregor J (2020). DirectPET: full-size neural network PET reconstruction from sinogram data. J Med Imaging.

[180] Liu Z, Ye H, Liu H (2022). Deep-learning-based framework for PET image reconstruction from sinogram domain. Appl Sci.

[181] Cui J, Zeng P, Zeng X, Wang P, Wu X, et al. TriDo-Former: A Triple-Domain Transformer for Direct PET Reconstruction from Low-Dose Sinograms. arXiv preprint arXiv:2308.05365.

[182] Hashimoto F, Ote K. ReconU-Net: a direct PET image reconstruction using U-Net architecture with back projection-induced skip connection. arXiv preprint arXiv:2312.02494. 2023. 10.1088/1361-6560/ad40f6 38640921

[183] Matej S, Surti S, Jayanthi S, Daube-Witherspoon ME, Lewitt RM, Karp JS (2009). Efficient 3-D TOF PET reconstruction using view-grouped histo-images: DIRECT—Direct image reconstruction for TOF. IEEE Trans Med Imaging.

[184] Whiteley W, Panin V, Zhou C, Cabello J, Bharkhada D, Gregor J (2021). FastPET: near real-time reconstruction of PET histo-image data using a neural network. IEEE Trans Radiat Plasma Med Sci.

[185] Feng T, Yao S, Xi C, Zhao Y, Wang R (2021). Deep learning-based image reconstruction for TOF PET with DIRECT data partitioning format. Phys Med Biol.

[186] Ote K, Hashimoto F (2022). Deep-learning-based fast TOF-PET image reconstruction using direction information. Radiol Phys Technol.

[187] Lv L, Zeng GL, Zan Y, Hong X, Guo M (2022). A back-projection-and-filtering-like (BPF-like) reconstruction method with the deep learning filtration from listmode data in TOF-PET. Med Phys.

[188] Gong K, Guan J, Kim K, Zhang X, Yang J (2019). Iterative PET image reconstruction using convolutional neural network representation. IEEE Trans Med Imaging.

[189] Boyd S, Parikh N, Chu E, Peleato B, Eckstein J (2011). Distributed optimization and statistical learning via the alternating direction method of multipliers. Found Trends Mach Learn.

[190] Zhang H, Goodfellow I, Metaxas D, Odena A. Self-Attention Generative Adversarial Networks. In: Proceedings of the 36th International Conference on Machine Learning, Proceedings of Machine Learning Research. 2019;97:7354–7363.

[191] Xie Z, Baikejiang R, Li T, Zhang X, Gong K (2020). Generative adversarial network based regularized image reconstruction for PET. Phys Med Biol.

[192] Gong K, Catana C, Qi J, Li Q (2019). PET image reconstruction using deep image prior. IEEE Trans Med Imaging.

[193] Ote K, Hashimoto F, Onishi Y, Isobe T, Ouchi Y (2023). List-mode PET image reconstruction using deep image prior. IEEE Trans Med Imaging.

[194] Cao X, Xie Q, Xiao P (2015). A regularized relaxed ordered subset list-mode reconstruction algorithm and its preliminary application to under-sampling PET imaging. Phys Med Biol.

[195] Hashimoto F, Ote K, Onishi Y (2022). PET image reconstruction incorporating deep image prior and a forward projection model. IEEE Trans Radiat Plasma Med Sci.

[196] Hashimoto F, Onishi Y, Ote K, Tashima H, Yamaya T (2023). Fully 3D implementation of the end-to-end deep image prior-based PET image reconstruction using block iterative algorithm. Phys Med Biol.

[197] Shan Q, Wang J, Liu D (2023). Deep Image Prior Based PET Reconstruction From Partial Data. IEEE Trans Radiat Plasma Med Sci.

[198] Chen S, Liu H, Shi P, Chen Y (2015). Sparse representation and dictionary learning penalized image reconstruction for positron emission tomography. Phys Med Biol.

[199] Mehranian A, Reader AJ (2021). Model-based deep learning PET image reconstruction using forward–backward splitting expectation–maximization. IEEE Trans Radiat Plasma Med Sci.

[200] Combettes PL, Pesquet JC. Proximal splitting methods in signal processing. In: Bauschke HH, Burachik RS, Combettes PL, Elser V, Luke DR, Wolkowicz H, editors. Fixed-Point Algorithms for Inverse Problems in Science and Engineering. New York, NY, USA: Springer; 2011. p. 185–212. doi: 10.1007/978-1-4419-9569-8_10.

[201] Kim K, Wu D, Gong K, Dutta J, Kim JH (2018). Penalized PET reconstruction using deep learning prior and local linear fitting. IEEE Trans Med Imaging.

[202] He K, Sun J, Tang X (2013). Guided image filtering. IEEE Trans Pattern Anal Mach Intell.

[203] Gong K, Wu D, Kim K, Yang J, Sun T, et al. MAPEM-Net: An unrolled neural network for Fully 3D PET image reconstruction. In: The 15th International Meeting on Fully Three-Dimensional Image Reconstruction in Radiology and Nuclear Medicine, Philadelphia, United States, 1107200, 2019. doi: 10.1117/12.2534904.

[204] Gong K, Wu D, Kim K, Yang J, El Fakhri G, et al. EMnet: an unrolled deep neural network for PET image reconstruction. In: SPIE Medical Imaging 2019: Physics of Medical Imaging, San Diego, California, United States, 1094853, 2019. doi: 10.1117/12.2513096.

[205] Lim H, Chun IY, Dewaraja YK, Fessler JA (2020). Improved low-count quantitative PET reconstruction with an iterative neural network. IEEE Trans Med Imaging.

[206] Xie N, Gong K, Guo N, Qin Z, Wu Z (2022). Penalized-likelihood PET image reconstruction using 3D structural convolutional sparse coding. IEEE Trans Biomed Eng.

[207] Hu R, Liu H. TransEM: Residual swin-transformer based regularized PET image reconstruction. In: International Conference on Medical Image Computing and Computer-Assisted Intervention. Cham: Springer Nature Switzerland; 2022. p. 184–193. doi: 10.1007/978-3-031-16440-8_18

[208] Xie H, Thorn S, Liu YH, Lee S, Liu Z (2022). Deep-Learning-Based Few-Angle Cardiac SPECT Reconstruction Using Transformer. IEEE Trans Radiat Plasma Med Sci.

[209] Li Z, Dewaraja YK, Fessler JA (2023). Training End-to-End Unrolled Iterative Neural Networks for SPECT Image Reconstruction. IEEE Trans Radiat Plasma Med Sci.

[210] Hu R, Chen Y, Kim K, Rockenbach MABC, Li Q, Liu H. DULDA: Dual-domain Unsupervised Learned Descent Algorithm for PET image reconstruction. arXiv preprint arXiv:2303.04661. 2023.

[211] Reader AJ. Self-Supervised and Supervised Deep Learning for PET Image Reconstruction. arXiv preprint arXiv:2302.13086. 2023.

[212] Liao S, Mo Z, Zeng M, Wu J, Gu Y (2023). Fast and low-dose medical imaging generation empowered by hybrid deep-learning and iterative reconstruction. Cell Rep Med.

[213] Zhang Q, Hu Y, Zhao Y, Cheng J, Fan W (2023). Deep Generalized Learning Model for PET Image Reconstruction. IEEE Trans Med Imaging.

[214] Shen C, Xia W, Ye H, Hou M, Chen H (2022). Unsupervised Bayesian PET Reconstruction. IEEE Trans Radiat Plasma Med Sci.

[215] Lv Y, Xi C (2021). PET image reconstruction with deep progressive learning. Phys Med Biol.

[216] Li J, Xi C, Dai H, Wang J, Lv Y (2023). Enhanced PET imaging using progressive conditional deep image prior. Phys Med Biol.

[217] Kamasak ME, Bouman CA, Morris ED, Sauer K (2005). Direct reconstruction of kinetic parameter images from dynamic PET data. IEEE Trans Med Imaging.

[218] Matthews J, Bailey D, Price P, Cunningham V (1997). The direct calculation of parametric images from dynamic PET data using maximum-likelihood iterative reconstruction. Phys Med Biol.

[219] Wang G, Qi J (2013). Direct Estimation of Kinetic Parametric Images for Dynamic PET. Theranostics.

[220] Yokota T, Kawai K, Sakata M, Kimura Y, Hontani H. Dynamic PET image reconstruction using nonnegative matrix factorization incorporated with deep image prior. In: 2019 IEEE/CVF International Conference on Computer Vision (ICCV), Seoul, Korea (South), 3126–3135, 2019. doi: 10.1109/ICCV.2019.00322.

[221] Wang B, Liu H (2020). FBP-Net for direct reconstruction of dynamic PET images. Phys Med Biol.

[222] Li S, Wang G (2022). Deep kernel representation for image reconstruction in PET. IEEE Trans Med Imaging.

[223] Hu R, Cui J, Yu C, Chen Y, Liu H. STPDnet: Spatial-temporal convolutional primal dual network for dynamic PET image reconstruction. arXiv preprint arXiv:2303.04667, 2023.

[224] Li Y, Hu J, Sari H, Xue S, Ma R, Kandarpa S (2023). A deep neural network for parametric image reconstruction on a large axial field-of-view PET. Eur J Nucl Med Mol Imaging.

[225] Wang X, Girshick R, Gupta A, He K. Non-local neural networks. In: Proceedings of the IEEE Conference on Computer Vision and Pattern Recognition. 2018:7794–7803.

[226] Gong K, Catana C, Qi J, Li Q (2021). Direct reconstruction of linear parametric images from dynamic PET using nonlocal deep image prior. IEEE Trans Med Imaging.

[227] Huang SC, Carson RE, Hoffman EJ, Kuhl DE, Phelps ME (1982). An investigation of a double-tracer technique for positron computerized tomography. J Nucl Med.

[228] Cheng X, Li Z, Liu Z, Navab N, Huang S-C, Keller U, Ziegler SI, Shi K (2015). Direct parametric image reconstruction in reduced parameter space for rapid multi-tracer PET imaging. IEEE Trans Med Imaging.

[229] Xu J, Liu H (2019). Deep-learning-based separation of a mixture of dual-tracer single-acquisition PET signals with equal half-lives: a simulation study. IEEE Trans Radiat Plasma Med Sci.

[230] Xu J, Liu H (2019). Three-dimensional convolutional neural networks for simultaneous dual-tracer PET imaging. Phys Med Biol.

[231] Qing M, Wan Y, Huang W, Xu Y, Liu H (2021). Separation of dual-tracer PET signals using a deep stacking network. Nucl Instrum Methods Phys Res A.

[232] Tong J, Wang C, Liu H (2022). Temporal information-guided dynamic dual-tracer PET signal separation network. Med Phys.

[233] Zeng F, Fang J, Muhashi A, Liu H (2023). Direct reconstruction for simultaneous dual-tracer PET imaging based on multi-task learning. EJNMMI res.

[234] Pan B, Marsden PK, Reader AJ (2023). Dual-Tracer PET Image Separation by Deep Learning: A Simulation Study. Appl Sci.

[235] Cherry SR, Jones T, Karp JS, Qi J, Moses WW, Badawi RD (2018). Total-body PET: maximizing sensitivity to create new opportunities for clinical research and patient care. J Nucl Med.

[236] Badawi RD, Shi H, Hu P, Chen S, Xu T, Price PM (2019). First human imaging studies with the EXPLORER total-body PET scanner. J Nucl Med.

[237] Wang Y, Li E, Cherry SR, Wang G (2021). Total-body PET kinetic modeling and potential opportunities using deep learning. PET clinics.

[238] Ultra-low Dose PET Imaging Challenge - Grand Challenge. https://ultra-low-dose-pet.grand-challenge.org/. Accessed 22 August 2023.

[239] Ota R (2021). Photon counting detectors and their applications ranging from particle physics experiments to environmental radiation monitoring and medical imaging. Radiol Phys Technol.

[240] Lecoq P, Morel C, Prior JO, Visvikis D, Gundacker S, et al. Roadmap toward the 10 ps time-of-flight PET challenge. Phys Med Biol. 2020;65(21):21RM01. 10.1088/1361-6560/ab9500 PMC7721485 32434156

[241] Ota R, Nakajima K, Ogawa I, Tamagawa Y, Shimoi H, et al. Coincidence time resolution of 30 ps FWHM using a pair of Cherenkov-radiator-integrated MCP-PMTs. Phys Med Biol. 2019;64(7):07LT01. 10.1088/1361-6560/ab0fce 30870825

[242] Ota R, Nakajima K, Ogawa I, Tamagawa Y, Kwon SI (2021). Lead-free MCP to improve coincidence time resolution and reduce MCP direct interactions. Phys Med Biol.

[243] Kwon SI, Ota R, Berg E, Hashimoto F, Nakajima K (2021). Ultrafast timing enables reconstruction-free positron emission imaging. Nat Photonics.

[244] Berg E, Cherry SR. Using convolutional neural networks to estimate time-of-flight from PET detector waveforms. Phys Med Biol. 2018;63(2):02LT01. 10.1088/1361-6560/aa9dc5 PMC5784837 29182151

[245] Onishi Y, Hashimoto F, Ote K, Ota R. Unbiased TOF estimation using leading-edge discriminator and convolutional neural network trained by single-source-position waveforms. Phys Med Biol. 2022;67(4):04NT01. 10.1088/1361-6560/ac508f 35100575

[246] Maebe J, Vandenberghe S (2022). Simulation study on 3D convolutional neural networks for time-of-flight prediction in monolithic PET detectors using digitized waveforms. Phys Med Biol.

[247] Hashimoto F, Ote K, Ota R, Hasegawa T (2019). A feasibility study on 3D interaction position estimation using deep neural network in Cherenkov-based detector: A Monte Carlo simulation study. Biomed Phys Eng Express.

[248] Ote K, Ota R, Hashimoto F, Hasegawa T (2020). Direct annihilation position classification based on deep learning using paired Cherenkov detectors: a Monte Carlo study. Appl Sci.

[249] He W, Zhao Y, Zhao X, Huang W, Zhang L (2023). A CNN-based four-layer DOI encoding detector using LYSO and BGO scintillators for small animal PET imaging. Phys Med Biol.

[250] Lee S, Lee JS (2023). Experimental evaluation of convolutional neural network-based inter-crystal scattering recovery for high-resolution PET detectors. Phys Med Biol.

