# Correction to: Deep learning-based PET image denoising and reconstruction: a review

**DOI:** 10.1007/s12194-024-00794-x

**Published:** 2024-03-16

**Authors:** Fumio Hashimoto, Yuya Onishi, Kibo Ote, Hideaki Tashima, Andrew J. Reader, Taiga Yamaya

**Affiliations:** 1 Central Research Laboratory, Hamamatsu Photonics K. K, 5000 Hirakuchi, Hamana-Ku, Hamamatsu, 434-8601 Japan; 2 Graduate School of Science and Engineering, Chiba University, 1-33, Yayoicho, Inage-Ku, Chiba, 263-8522 Japan; 3 National Institutes for Quantum Science and Technology, 4-9-1, Anagawa, Inage-Ku, Chiba, 263-8555 Japan; 4 School of Biomedical Engineering and Imaging Sciences, King’s College London, London, SE1 7EH UK

**Correction to: Radiological Physics and Technology (2024) 17:24–46** 10.1007/s12194-024-00780-3

In the original PDF of this article, the formula 5 was published with extraneous text as “* 20*c*”.

Incorrect version:

5 $$* 20cL\left({\varvec{y}}|{\varvec{x}}\right)=-{\text{log}}P\left({\varvec{y}}|{\varvec{x}}\right)=C-{\sum }_{i=1}^{I}\left\{{y}_{i}{\text{log}}\left({\sum }_{j=1}^{J}{a}_{\mathit{ij}}{x}_{j}+{\overline{b} }_{i}\right)-\left({\sum }_{j=1}^{J}{a}_{ij}{x}_{j}+{\overline{b} }_{i}\right)\right\},$$

Correct version:

5 $$L\left({\varvec{y}}|{\varvec{x}}\right)=-{\text{log}}P\left({\varvec{y}}|{\varvec{x}}\right)=C-{\sum }_{i=1}^{I}\left\{{y}_{i}{\text{log}}\left({\sum }_{j=1}^{J}{a}_{\mathit{ij}}{x}_{j}+{\overline{b} }_{i}\right)-\left({\sum }_{j=1}^{J}{a}_{ij}{x}_{j}+{\overline{b} }_{i}\right)\right\},$$

The HTML version was unaffected.

In this article the formula 13 was incorrectly published with a minus (-) symbol in the denominator instead of plus ( +) symbol.

Incorrect version:

13 $${x}_{j}^{\left(k+1\right)}=\frac{{x}_{j}^{\left(k\right)}}{\sum_{i=1}^{I}{a}_{ij}-\beta {\left.\frac{\partial U\left({\varvec{x}}\right)}{\partial {x}_{j}}\right|}_{{\varvec{x}}={{\varvec{x}}}^{(k)}}}{\sum }_{i=1}^{I}\frac{{a}_{ij}{y}_{i}}{{\sum }_{{j}^{\prime}=1}^{J}{a}_{i{j}^{\prime}}{x}_{{j}^{\prime}}^{\left(k\right)}+{\overline{b} }_{i}}.$$

Correct version:

13 $${x}_{j}^{\left(k+1\right)}=\frac{{x}_{j}^{\left(k\right)}}{\sum_{i=1}^{I}{a}_{ij}+\beta {\left.\frac{\partial U\left({\varvec{x}}\right)}{\partial {x}_{j}}\right|}_{{\varvec{x}}={{\varvec{x}}}^{(k)}}}{\sum }_{i=1}^{I}\frac{{a}_{ij}{y}_{i}}{{\sum }_{{j}^{\prime}=1}^{J}{a}_{i{j}^{\prime}}{x}_{{j}^{\prime}}^{\left(k\right)}+{\overline{b} }_{i}}.$$

The original article has been corrected.

